# Tuning the Bulk and Surface Properties of PDMS Networks through Cross-Linker and Surfactant Concentration

**DOI:** 10.1021/acs.macromol.1c01600

**Published:** 2021-10-06

**Authors:** Matthew Litwinowicz, Sarah Rogers, Andrew Caruana, Christy Kinane, James Tellam, Richard Thompson

**Affiliations:** † Department of Chemistry, Durham University, Durham DH1 3LE, United Kingdom; ‡ STFC ISIS Facility, Rutherford Appleton Laboratories, Chilton, Didcot OX11 0QX, United Kingdom

## Abstract

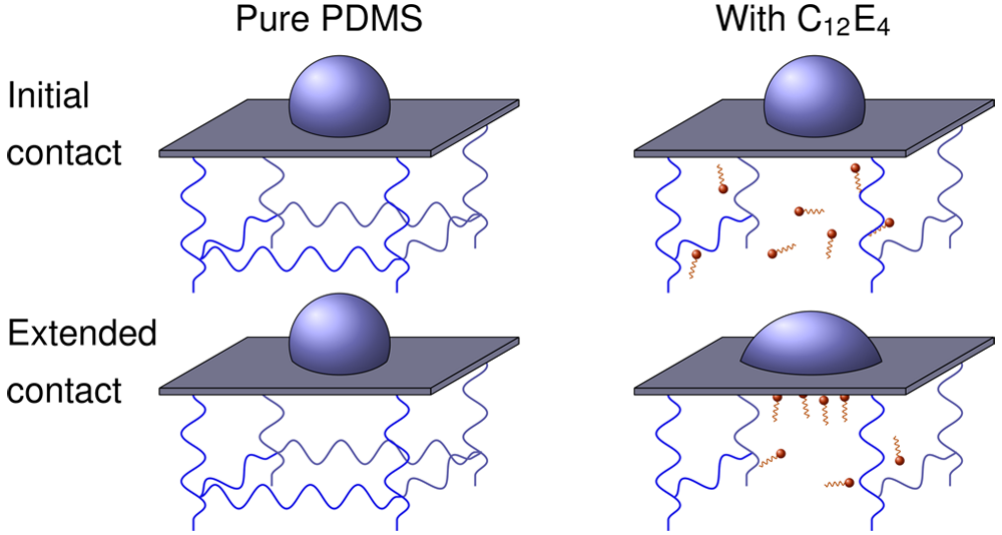

The elastic modulus and hydrophilicity of cross-linked poly(dimethylsiloxane) (PDMS) are tunable via cross-linker concentration and the addition of a simple surfactant, C_12_E_4_, before curing. However, the surfactant concentration, [C_12_E_4_], reduces the elastic modulus (73% lower for 6.3% w/w) because it reduces the extent of curing. This is likely because the hygroscopic surfactant results in water poisoning of the catalyst. Three distinct time-dependent hydrophilicity profiles were identified using water contact angle analysis with [C_12_E_4_] determining which profile was observed. This indicates the concentration-dependent phase behavior of C_12_E_4_ within PDMS films. Changes in phase behavior were identified using small-angle neutron scattering (SANS) and a compatibility study. No surface excess or surface segregation of surfactant was observed at the PDMS–air interface. However, a surface excess revealed by neutron reflectivity against a D_2_O interface indicates that the increase in hydrophilicity results from the migration of C_12_E_4_ to the film interface when exposed to water.

## Introduction

Poly(dimethylsiloxane) (PDMS) is a polymer that has use in microfluidic devices,^1−4^ fouling-release coatings,^5−9^ and cell cultivation.^10,11^ When cross-linked to form a network, its biocompatibility, low bulk modulus, deformability, low roughness, and low surface energy have made the polymer a favorable material for these applications,^12^ and by learning to better control some of these properties, the functionality of PDMS in these applications can be improved.

However, PDMS does have limitations. For example, the hydrophobicity of the coating allows oils and proteins to nucleate on the surface, one of the short time-scale events in fouling.^13^ Limiting this fouling is a key issue for the long-term efficacy of antifouling coatings on marine vessels, as well as for microfluidic devices.^1,6^ Methods that have been explored previously include the chemical modification of PDMS with hydrophilic moieties and the addition of surfactants to the precure PDMS mixture,^14−19^ resulting in hydrophilic surfaces with improved resistance to nonspecific protein adsorption and biofouling.

This hydrophobicity also mandates the use of an external pumping system in a microfluidic device. Holczer and Fürjes sought to improve the hydrophilicity of PDMS to allow a microfluidic device to be self-driven.^2^ This was done by adding surfactants to the PDMS before curing, resulting in improved transport efficiency through microfluidic channels, demonstrating the benefits of improving the hydrophilicity of PDMS.

In the case of cell cultivation and tissue engineering, it has been found that matching the elastic modulus of the scaffold to the tissue enhances the proliferation of cells.^20,21^ Cameron et al. reported that, for 2D and 3D scaffolds, the improved proliferation of bladder cells could be achieved when the elastic modulus of the poly(lactide-*co*-glycolide) and poly(ϵ-caprolactone) scaffolds were closer to that of bladder tissues. PDMS has been investigated as a 3D tissue scaffold due to its biocompatibility, ease of processing, and low elastic modulus.^10,11^

In addition, the migration or release of surfactants may have a role in the development of antimicrobial surfaces, with surfactants playing a role in artificial antiviral surfaces.^22^ Nonionic surfactants have been shown to have antimicrobial properties,^23^ with some ethoxylate surfactants showing specific efficacy against *Escherichia coli*.^24^ Films composed of dried dishwashing detergents have demonstrated virucidal activity against avian coronavirus,^25^ showing the potential benefit of a coating capable of exposing microbes to surfactants.

The cross-link density of a polymer network can be altered by changing the initial concentration of cross-linker in the elastomer base. The cross-link density, ν, is related to the elastic modulus of a rubber, *G*_R_^′^, by the relation^26^
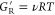
1where *R* is the universal gas constant, and *T* is the temperature. As such, controlling the cross-link density allows for a degree of control over the elastic modulus. Previous research has evaluated the elastic modulus in commercial PDMS Sylgard 184.^27−32^ Wang et al. varied the ratio of elastomer base to cross-linker from 5:1 to 33:1 and used a compression test to determine the elastic modulus.^28^ The group found that, as expected, a higher concentration of cross-linker resulted in a greater elastic modulus and produced results similar to previous studies, which used a mixture of compression and tension tests to find the modulus.^33,34^ This research group later compared microscale and macroscale methods of obtaining the elastic modulus for PDMS, concluding that good agreement was possible, provided the method was capable of accounting for the adhesion between a probe and the PDMS surface at low cross-link densities.^29^ However, Johnston et al. have highlighted a possible dependence of the elastic modulus on the temperature at which PDMS is cured.^27^ Depending on the technique used, either the shear modulus, *G*, or Young’s modulus, *E*, may be measured. However, these two moduli are related by^35^
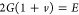
2where *v* is Poisson’s ratio for a material, and *v* is typically near 0.5 for similar materials, such as rubber.^36^ As such, measuring one will indicate the trend of the other as well.

We postulate that, in addition to modifying surface properties, the incorporation of a nonionic surfactant to the matrix may have an impact on the bulk properties. The extent to which these important properties are coupled has received little attention. Some groups have suggested that the change in the bulk properties of polymers following the small addition of surfactants is limited,^4,5,37,38^ whereas other research has discussed the existence of changes to bulk properties.^2,39^ With uncertainty in the effect of surfactants on bulk polymer properties, it is important to understand these relationships on a quantitative level.

Nonionic surfactants have previously been shown to increase the hydrophilicity of a PDMS surface following exposure to water.^2,5,6,40,41^ Water contact angle analysis (WCA), namely, the sessile drop method, has demonstrated initial contact angles of over 90° for both pure PDMS films and also surfactant/PDMS films. A contact angle greater than 90° is indicative of a hydrophobic surface. However, when left over time, the contact angle, θ, for surfactant/PDMS films has been shown to decrease to below 90°, indicating a hydrophilic surface.^5,39−41^ Conversely, the static contact angle is greater than 90° for pure PDMS films.^2,40^

The Young equation can be expressed as^42^
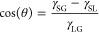
3where γ_SG_, γ_SL_, and γ_LG_ are the solid–gas, solid–liquid, and liquid–gas interfacial energies, respectively. Critically, for θ to switch from above to below 90°, it is necessary for a switch in condition from γ_SG_ < γ_SL_ to γ_SG_ > γ_SL_. For an initially dry film placed in contact with a water droplet, this implies a large reduction in γ_SL_ following contact between PDMS and water and thus an increase in the hydrophilicity of the film’s surface.

It has been suggested that this reduction in the solid–liquid interfacial energy is the result of a migration of surfactant molecules through the PDMS matrix to the surface.^2,40^ Following this migration, the hydrophilic moieties would be exposed to water, reducing the hydrophobic interactions between the PDMS surface and water. As such, this migration would be driven by a reduction in the interfacial energy. This migration has been used to find the diffusion coefficients, *D*, of surface-active migrants in PDMS by Camós Noguer et al.^5^ This method consists of measuring the time taken for a decrease in water contact angle to be measured following migration from surfactant-doped PDMS through an undoped PDMS film. This time lag can be used to find a diffusion coefficient assuming Fickian diffusion. The most significant factor affecting *D* was found to be the molecular weight, *M*_w_, of the surfactant. However, in spite of this, *D* was found to have limited importance in determining the quality of fouling-release properties observed.

Fatona et al. found that the addition of nonionic surfactants to PDMS resulted in observable features at the PDMS interface using atomic force microscopy (AFM), increasing the surface roughness.^43^ Roughness is a concern as it is a characteristic that can negatively affect the fouling-release properties of a coating due to the improved ability of fouling species to adhere when larger surface features are present.^9,44^ In addition, swelling of surface features has been observed when surfaces were exposed to water.^43,45^ This demonstrates not only that surfactant may be present at the PDMS–air interface but that the surface restructures in contact with water.

While surface restructuring is likely important for hydrophilicity and roughness, migration of the surfactants when the PDMS is in contact with water likely also has a significant role in these characteristics. Camós Noguer et al. used confocal laser scanning microscopy (CLSM) to observe that a surface excess of a PEO–PDMS–PEO surfactant existed when a surfactant/PDMS film was placed in water and that this surface excess increased with immersion time in water. This is evidence of a migration of a surfactant from the bulk to the interface and suggests the importance of understanding the vertical concentration profile of a surfactant in a PDMS film. In addition, the experiment revealed the presence of large surfactant domains (≤7 μm) in the bulk of the PDMS due to poor compatibility.

Compatibility is an important factor to consider: if components are incompatible, the clarity of the material, which is usually desirable in microfluidic devices,^40^ may be reduced. Alternatively, the presence of large phase-separated domains may result in a high surface roughness. The incompatibility of components in a polymeric system has also been found to contribute to the surface segregation of surfactants or the development of a wetting layer, as observed by Briddick et al. for ionic and nonionic surfactants in poly(vinyl alcohol).^46,47^

This article explores the extent to which the hydrophilicity and elastic modulus of commercially available cross-linkable PDMS, Sylgard 184, can be tuned by changing the concentration of cross-linker used in curing and the addition of a simple surfactant, tetraethylene glycol monododecyl ether (C_12_E_4_). By providing information on the tunability of these characteristics and causes behind the observed effects, this work will aid in formulation by the design of fouling-release coatings, among other applications, using low-cost and scalable methods.

## Experimental Section

### Materials and Sample Preparation

**Figure 1:**
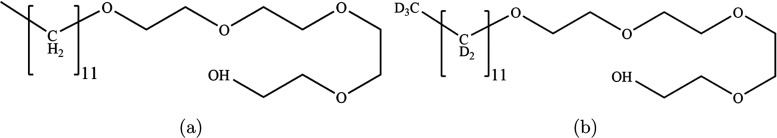
Structures of the molecules C_12_E_4_ (a) and d_25_–C_12_E_4_ (b).

Sylgard 184 silicone elastomer (Dow Corning) was obtained from Ellsworth Adhesives, U.K., and the two parts were mixed in defined proportions to produce PDMS samples. C_12_E_4_ (Sigma-Aldrich) was used as received, while d_25_–C_12_E_4_ was synthesized at the ISIS Deuteration Facility.^48^ The structures of C_12_E_4_ and d_25_–C_12_E_4_ are shown in Figure 1.

The recommended mixing ratio of Sylgard 184 is 10:1 part A to part B,^49^ where part B is the cross-linker. This ratio was used for most experiments discussed in this work, although altering this ratio was explored in others. The concentration of part B is stated throughout and is given as a weight percentage.

Following mixing of the Sylgard 184 components, C_12_E_4_ (or d_25_–C_12_E_4_ for small-angle neutron scattering (SANS), neutron reflectivity (NR), and NRA experiments) was added and mixed. The samples were left at room temperature to allow air bubbles to be removed. In the case of higher viscosity mixtures, samples were placed under vacuum to aid in air bubble removal.

For experiments requiring films, the C_12_E_4_/PDMS mixtures were spin-cast without solvent onto silicon wafer for 1 min at 5000 rpm and were cured in an oven at 120 °C for at least 2 h to ensure curing completion.

### In Situ Cure in a Rheometer

An AR2000 (TA Instruments) rheometer was used for measurements. Samples were loaded uncured into an 8 mm parallel plate (1 mm gap) geometry and enclosed within an environmental test chamber (ETC). This geometry was chosen due to the large variation in stress response expected throughout the course of the experiment, allowing precise measurements to be carried out on a single sample in both the cured and uncured states.

Samples were heated from 30 to 120 °C in a typical curing reaction. In the case that the PDMS had not cured, the sample was heated to higher temperatures until cured. Oscillatory measurements were made at 1 Hz at intervals of 1 °C, allowing 1 min for equilibration once the temperature was reached. The effective average heating rate was 0.47 °C min^–1^. Typical data are given in Figure S.1. By observing the changes in the storage and loss moduli, *G*′ and *G*″, respectively, with temperature, the curing profile could be observed.

Following curing, a frequency sweep from 0.1 to 100 Hz using 5 points per decade was performed at 140 °C. The plateau modulus was found by taking the mean of *G*′ across the frequency range 0.1–0.25 Hz, where the modulus was largely consistent.

### Atomic Force Microscopy

AFM images were recorded using a Bruker MM8 AFM. The PeakForce tapping technique was performed on cured PDMS films with varying concentrations of C_12_E_4_, producing height maps, as well as adhesion maps and other surface properties of the films. The AFM probes used were ScanAsyst-Fluid, Bruker. These probes were selected due to their low spring constant, *k* = 0.7 N m^–1^, as stiffer probes resulted in damage to films.

Images were recorded with a resolution of 512 lines following curing, and then samples were immersed in UHP water for 24 h. Following immersion, a nitrogen stream was used to dry the surfaces before being scanned again. Scans were taken over an area of 20 μm × 20 μm. Gwyddion analysis software was used to process and analyze the images, with images being leveled using second-order polynomials. The root-mean-square surface roughness, *R*_q_, was determined using the equation^50^
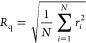
4where *N* is the number of data points and *r*_*i*_ is the deviation in height of a point from the mean.

### Nuclear Reaction Analysis

Nuclear reaction analysis (NRA) was used to produce vertical concentration profiles of d_25_–C_12_E_4_ in PDMS films under vacuum. To prevent the loss of d_25_–C_12_E_4_ under vacuum, samples were cooled in liquid nitrogen before being placed into the vacuum chamber.

By labeling the surfactant with deuterium and using a ^3^He source, the nuclear reaction^51^
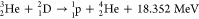
5will occur. The measured energy of a scattered proton is dependent on the depth in the film at which the reaction occurs, allowing a vertical concentration profile to be generated. The ^3^He^+^ beam energy was fixed at 0.7 MeV. Using this setup, a depth resolution of ∼8 nm can be obtained.^47^ This resolution is sufficient to evaluate the presence of a surface excess but is not sufficient for structural detail at the molecular scale of the surfactants. Greater detail on ion beam analysis, including NRA, can be found elsewhere.^52^

DataFurnace (Surrey University, WiNDF v9.3.68 running NDF v9.6a)^53^ was used to produce appropriate model fits for the resulting data sets. Due to the thickness of the films exceeding the range of the incident beam, the PDMS matrix was used to define the substrate.

### Water Contact Angle Analysis

Videos of changing water contact angles were recorded using a UI-3370CP-M-GL Rev.2 camera (IDS Imaging Development Systems). Five microliters of UHP water was placed on PDMS films containing various concentrations of C_12_E_4_ and were recorded for 15 min. Video frames were extracted at an appropriate sampling rate, and contact angles were measured using DropSnake from the Drop Shape Analysis package on ImageJ.^54^ The frame sampling rate was 0.067–15 fps, depending on the rate of the change of droplet shape.

### Neutron Reflectivity

Neutron reflectivity (NR) is a technique capable of producing high-resolution (<1 nm) vertical concentration profiles of deuterated materials in polymers^55−57^ and has previously been used to detect blooming of amphiphiles in poly(vinyl alcohol) films.^46,47,58^ NR has the advantage of being operable under a variety of experimental conditions, whereas similar concentration profiling techniques, such as NRA, elastic recoil detection analysis (ERDA), and Rutherford backscattering (RBS), are required to operate under vacuum. With its previous application in detecting surface enrichment in surfactant/polymer systems and its ability to operate under a variety of experimental geometries and conditions, the technique is an excellent candidate for obtaining concentration profiles of surfactant/PDMS films both against an air and a water interface.

NR was performed on the POLREF reflectometer at the ISIS Pulsed Neutron Source (STFC Rutherford Appleton Laboratory, Didcot, U.K.).^59^ NR uses the interference of neutrons between layers of varying scattering length density (SLD or ρ) to produce a curve of reflectivity, *R*, against the scattering wavevector, *Q*. By building a model of different SLD layers and fitting the calculated reflectivity curve to the NR data, the vertical concentration profile of molecular components can be obtained. To obtain sufficient SLD contrast, d_25_–C_12_E_4_ was used in place of hydrogenous C_12_E_4_.

As NR can be performed under atmospheric pressure, a wider range of experimental setups were used than were possible with NRA. Reflectivity curves were obtained for d_25_–C_12_E_4_/PDMS films against an air interface, followed by the curve for films against a D_2_O interface. For the second measurement, D_2_O was placed onto a roughened silicon block. The d_25_–C_12_E_4_/PDMS film-coated blocks were then inverted and placed on top of the D_2_O. This geometry was chosen since it would put the D_2_O as the lowest layer, allowing a sharp critical edge to be produced by the total internal reflection of neutrons against the high SLD D_2_O layer (ρ_D_2_O_ = 6.37 × 10^–6^ Å^–2^).

To prepare the samples for NR, d_25_–C_12_E_4_ was mixed with Sylgard 184 part A and part B. Films were then produced by dissolving mixtures in hexane and spin-casting them onto silicon blocks. Films were cured at ∼100 °C for 1 h on a hot plate before measurement. For the measurements against the D_2_O interface, films were given sufficient time to equilibrate (>1 h) during the NR alignment procedure.

Although the local root-mean-square roughness measured over a micron-scale area by AFM was small, films had a gradual variation in thickness, which was treated separately from the layer roughness in the optical matrix calculation. This is necessary to consistently fit the shape of the critical edge in *R*(*Q*) along with the damping of the Kiessig fringes. An in-house NR fitting program was written, MUSCtR v1.4,^60^ which allowed the PDMS layer to be modeled as having an undulating thickness. The reflectivity calculation procedure is outlined in Figure S.2.

### Compatibility Testing

Samples of C_12_E_4_ in Sylgard 184 part A, or in a 10:1 mixture of Sylgard 184 part A:part B, were heated to 100 °C, and the turbidity was evaluated. The temperature was then decreased to 20 °C in increments of 5–10 °C, and the turbidity was evaluated following equilibration at each temperature. Turbidity of samples was categorized as high, medium, or none if they appeared fully turbid, translucent, or transparent, respectively.

### Small-Angle Neutron Scattering

Small-angle neutron scattering (SANS) was carried out on the Sans2d small-angle diffractometer at the ISIS Pulsed Neutron Source (STFC Rutherford Appleton Laboratory, Didcot, U.K.).^59,61^ SANS is a well-established technique for observing the structure in a system on the nanometer to micrometer scale.^62^

A simultaneous *Q*-range of 0.0015–0.25 Å^–1^ was achieved, utilizing an incident wavelength range of 1.75–12.5 Å and employing an instrument setup of L1 (source-to-sample distance) = L2 (sample-to-detector distance) = 12 m, with the 1 m^2^ detector offset vertically 60 mm and sideways 100 mm. *Q* is defined as
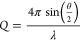
6where θ is the scattered angle and λ is the incident neutron wavelength. The beam diameter was 6 mm. Each raw scattering data set was corrected for the detector efficiencies, sample transmission, and background scattering and converted to scattering cross-sectional data ( vs *Q*) using the instrument-specific software.^63^ These data were placed on an absolute scale (cm^–1^) using the scattering from a standard sample (a solid blend of hydrogenous and perdeuterated polystyrene) in accordance with established procedures.^64^

The difference in SLD between PDMS (ρ_PDMS_ = 6 × 10^–8^ Å^–2^) and C_12_E_4_ (ρ_C_12_E_4__ = 7 × 10^–8^ Å^–2^) does not allow for sufficient contrast using SANS. As such, deuterium-labeled d_25_–C_12_E_4_ (ρ_d_25_–C_12_E_4__ = 4.2 × 10^–6^ Å^–2^) was used, allowing for much greater contrast with the PDMS.

Samples were prepared in the concentration range of ∼1–7% w/w d_25_–C_12_E_4_ in Sylgard 184 part A. These mixtures were then combined with Sylgard 184 part B (in a ratio of 10:1 A:B) and cured in quartz cells with a path length of 1 mm. The mixtures without part B were also loaded into quartz cells. SANS measurements were then taken for both sets of samples at 30, 60, and 90 °C. Data were then fit using SasView.^65^

## Results and Discussion

### Dependence of the Shear Modulus on the Concentration of Cross-Linker

**Figure 2:**
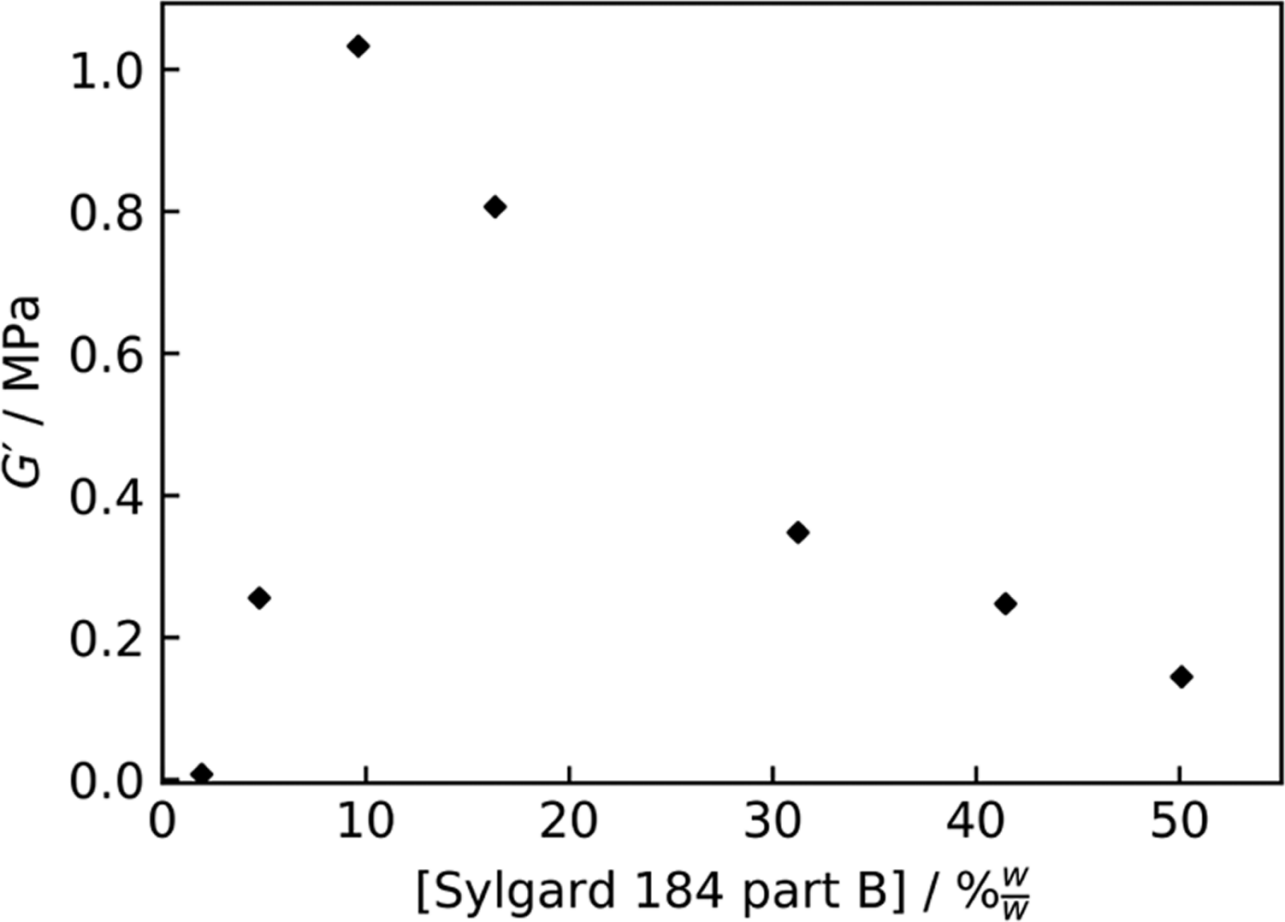
Shear storage modulus of cured PDMS networks changing with the concentration of Sylgard 184 part B. Data are from postcure frequency sweeps at a temperature of 140 °C.

The change in shear storage modulus of the cured PDMS against the concentration of cross-linker is shown in Figure 2. The plot shows a significant dependence of the shear modulus on the concentration of cross-linker used. In particular, there is a peak in the shear modulus near 10% w/w part B—the manufacturer’s recommendation would yield [Sylgard 184 part B] = 9.1% w/w. It is interesting to note that, at low concentrations of part B, the low modulus was expressed as a deformable, rubbery behavior, whereas at high concentrations, the resulting networks also had a low shear storage modulus but were brittle. This indicates a difference in the nonlinear rheology between the two regimes, despite the similar shear moduli. Similar observations regarding the trend in the tangent elastic modulus with cross-linker concentration and the different nonlinear rheologies have been reported by Seghir and Arscott when curing Sylgard 184 for 2 h at 100 °C.^66^

The reduction in *G*′ on either side of the peak at ∼10% w/w part B is significant, with ∼50% w/w part B resulting in a *G*′, which is 14% of the peak value, while ∼2% w/w yields a *G*′ only 0.7% of the peak modulus. This reduction on either side of the peak is indicative of a large proportion of reactions between part A and part B molecules failing to contribute to the cross-linked network but instead forming branching or uncoupled segments in the system. For example, with only two vinyl terminuses on a part A molecule capable of reacting through hydrosilylation^67^ (the reaction scheme can be seen in Figure S.3), it becomes less likely that a part A molecule will be capable of bonding to two different part B molecules at low [part B].

### Elastic Modulus of Networks Containing C_12_E_4_

**Figure 3:**
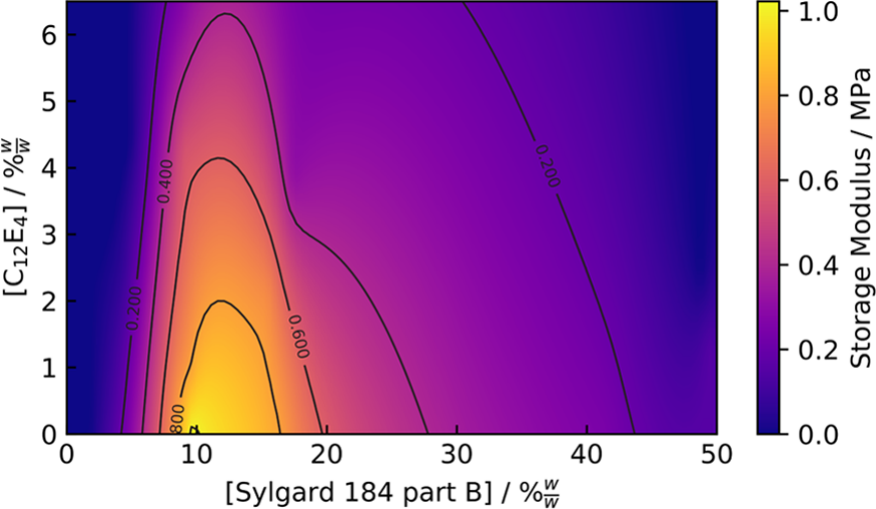
Effect on *G*′ of the cured PDMS when varying the concentration of cross-linker, as well as the C_12_E_4_ concentration. Measurements were made at 140 °C.

The plateau modulus was also measured for PDMS when varying the C_12_E_4_ concentration, as well as the concentration of cross-linker. A contour plot is shown in Figure 3.

The presence of C_12_E_4_ reduces the storage modulus of PDMS. We observe a ridge-like feature along the surface in the range 9–15% w/w part B. However, the reduction in *G*′ along this ridge is significant. With only a small reduction in the volume fraction of 10:1 part A:part B PDMS from 0% w/w C_12_E_4_, we find that the measured *G*′ at 6.3% w/w C_12_E_4_ is reduced by 73%, a much poorer cross-link yield than in the absence of C_12_E_4_, demonstrating that the effect of the surfactant is more than can be accounted for by plasticization and the “dilution” of the cross-links alone. This trend can also be observed at different concentrations of cross-linkers. From this, it would appear that the concentration of C_12_E_4_ must be considered if aiming for a specific elastic modulus. Previous studies have indicated that low surfactant concentrations have minimal effects on the bulk properties of polymers, including PDMS.^4,5,37,38^ Interestingly, Kim et al. observed an increase in Young’s modulus for PDMS when doped with a trisiloxane ethoxylate surfactant,^39^ demonstrating that the choice of surfactant may be important in determining the effect on the elastic modulus.

We postulate that the hygroscopic nature of C_12_E_4_ contributes to this effect by introducing water to the precured resin. It is possible that water, and indeed the −OH group on the surfactant itself, could offer a reaction route in competition with the cross-linking hydrosilylation for the Si–H bonds on the cross-linker.^68^ However, since NMR results show no new hydrogen or carbon environments following the mixing and heating of C_12_E_4_ and Sylgard 184 part B, it is more likely that water is poisoning the catalyst, rather than offering a competing reaction. This reaction scheme and the NMR analysis are included in Figures S.3 and S.4.

### Roughness Increases with C_12_E_4_ Concentration

In the absence of C_12_E_4_, AFM scans showed smooth spin-cast cured PDMS films with *R*_q_ < 2 nm and no distinct features on the μm scale. Once C_12_E_4_ was incorporated into the matrix and cured, bumps were detected on the surface on the order of a few micrometers in the *x* and *y* directions and <100 nm in the *z* direction. These features may be a result of phase separation or blooming of the surfactant at the interface.

**Figure 4:**
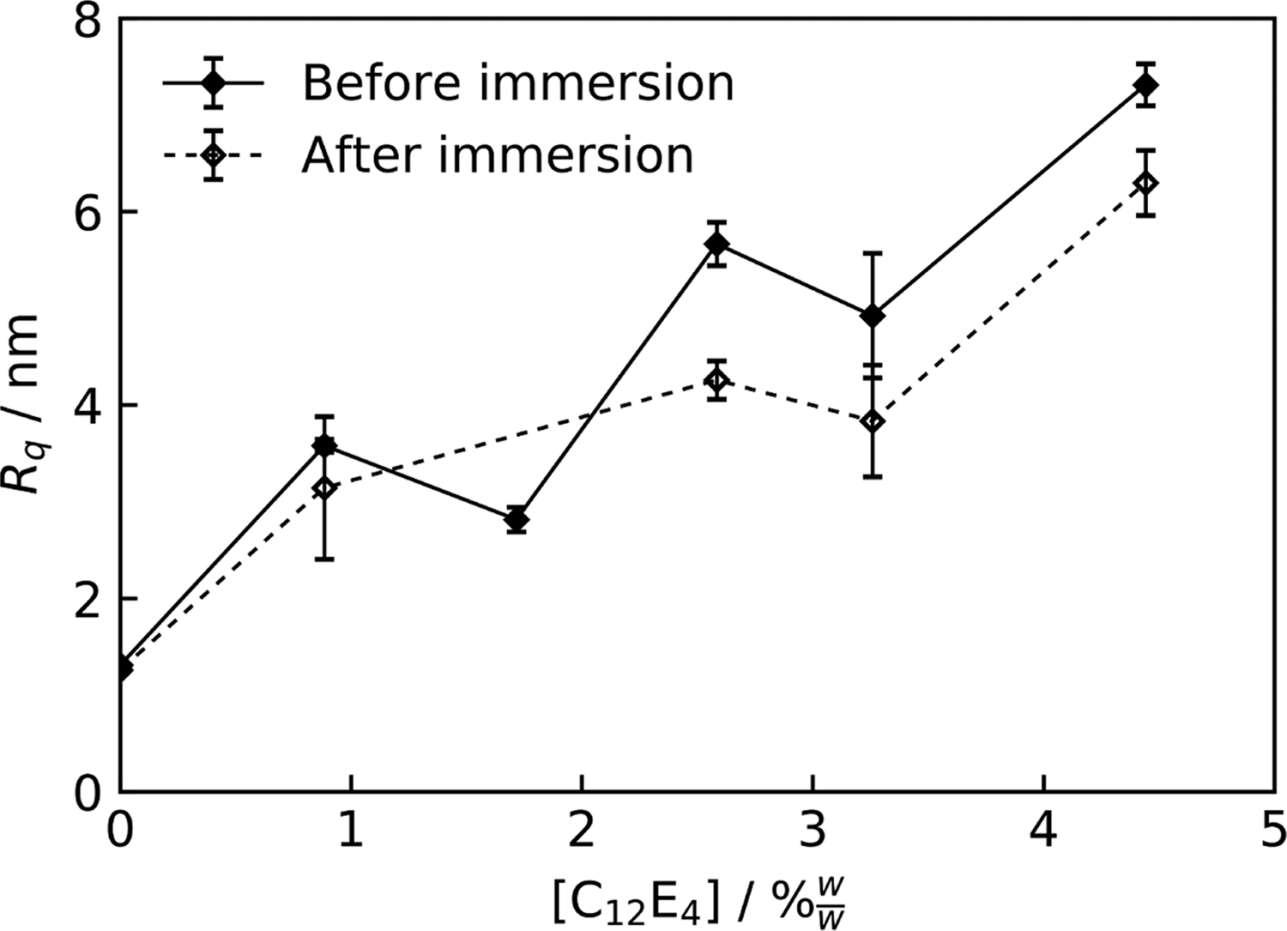
Surface roughness of C_12_E_4_/PDMS films before and after immersion in UHP water. The standard errors on the data points have been included, with lines connecting the data series to guide the eye.

We can consider the effect of C_12_E_4_ on surface features quantitatively using *R*_q_, found in eq 4. The resulting plot is shown in Figure 4. The plot shows that, both before and after immersion in water, an increase in the concentration of surfactant results in an increase in surface roughness. Example AFM scans are shown in Figure S.5. These images seem to suggest that the features grow in number and size with greater surfactant concentrations.

There does not appear to be a significant change when comparing scans before and after immersion in water (Figure 4), neither by visual inspection of the images nor by comparing their *R*_q_. This would initially suggest that the features are not surface-segregated surfactant, as removal of the surfactant, or other structural changes such as swelling, would be expected following immersion. Within the time scale of AFM measurements, dry PDMS samples showed little evidence of surfactant adsorption.

### Homogeneous Dispersion of C_12_E_4_ Near Air Interface

Using AFM, we were able to extract information on the adhesion between the silicon nitride probe and the material surface. The measured adhesion would be expected to have the sensitivity to resolve surfactants and polymers. Thus, if the features seen on AFM images were surfactant domains on the surface, we would expect there to be a strong correlation between the adhesion heat map and the height heat map.

**Figure 5:**
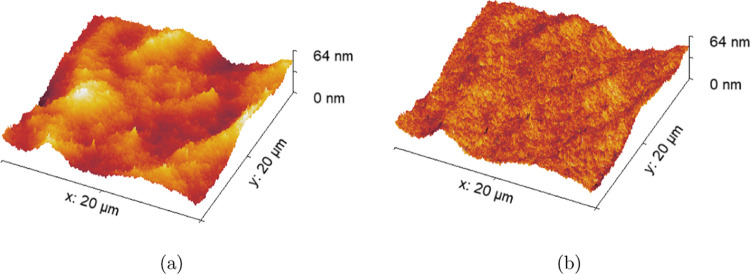
AFM height surface plots of a C_12_E_4_/PDMS film containing 4.4% w/w C_12_E_4_ with (a) a height heat map overlay and (b) an adhesion heat map overlay.

Figure 5 compares two height projections of a film of 4.4% w/w C_12_E_4_ in PDMS: one with a heat map overlay of the height and the other with a heat map overlay of the adhesion. It can be seen that there is little correlation between the two heat maps. The only features of the adhesion heat map appear to be a “shadowing” effect on the left-hand side of the height features in the *x* direction—this is likely due to the difference in contact area between the surface and probe when moving along a rising edge, as opposed to a falling edge, yielding this “shadow”. Based on the lack of correlation, there is no clear lateral variation in surface composition and thus no evidence for localized surfactant aggregates at the air interface.

**Figure 6:**
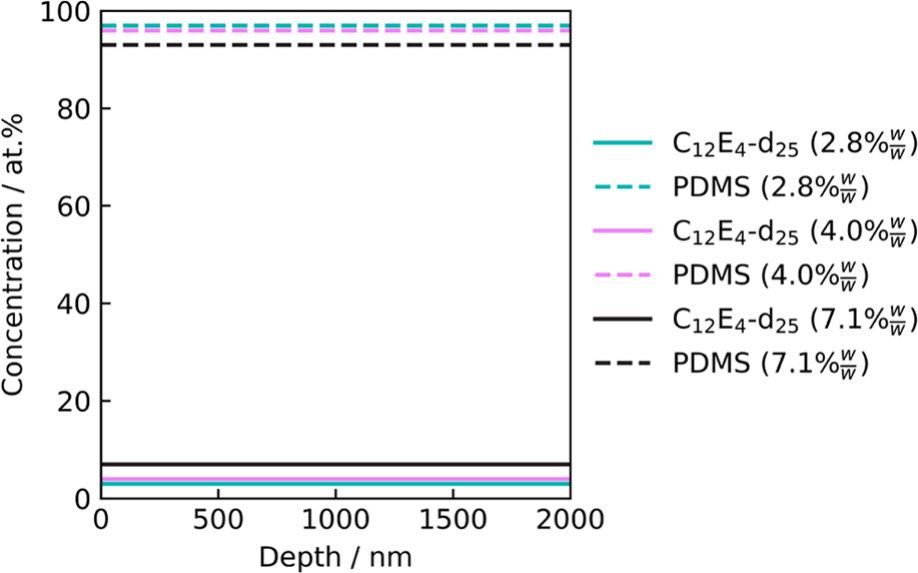
Vertical concentration profiles corresponding to the generated fits for NRA data for films of d_25_–C_12_E_4_/PDMS using a ratio of 10:1 Sylgard 184 part A:part B. A density of 1.01 g mL^–1^ was assumed for d_25_–C_12_E_4_. The raw NRA data and their fits are shown in Figure S.6.

NRA was performed on films of varying concentrations of d_25_–C_12_E_4_ in cured PDMS to verify this observation. The resulting depth profiles are shown in Figure 6. The depth profiles at all three concentrations (2.8% w/w, 4.0% w/w, and 7.1% w/w d_25_–C_12_E_4_) revealed a homogeneous distribution of the deuterated surfactant throughout the depth of the films. This is surprising when compared to other surfactant/polymer systems, which have shown a surface excess of surfactant.^46,47,69^ However, this difference can be justified by the lower surface energy of PDMS than other polymers, such as poly(vinyl alcohol). This result is also consistent with the lack of contrast in adhesion at the surface observed using AFM—surface-segregated, or surface-enriched, surfactant would not reveal a homogeneous depth profile.

### Water Exposure Triggers a Hydrophobic-to-Hydrophilic Switch at the Interface

**Figure 7:**
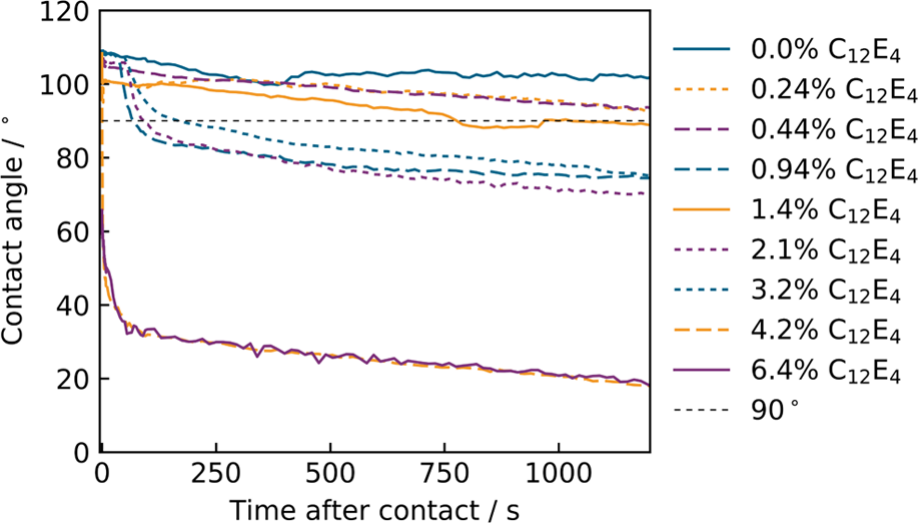
Time-resolved WCA on films of varying C_12_E_4_ concentrations in PDMS. PDMS was prepared with a 10:1 ratio of Sylgard 184 part A:part B. A line is included at θ = 90° to highlight the hydrophobic–hydrophilic boundary.

Figure 7 shows clear evidence of hydrophobic–hydrophilic switching behavior when C_12_E_4_ is incorporated in the PDMS and then exposed to water. The behavior of C_12_E_4_ is therefore representative of the phenomenology reported elsewhere for other surfactants in PDMS.^2,5,6,40,41^

Films at all investigated C_12_E_4_ concentrations began with contact angles greater than 90°. AFM revealed a negligible change in the rugosity factor between films and thus will not have an effect on the initial contact angle. The water contact angle dropped below 90° within a 2 min period for all films containing >0.94% w/w C_12_E_4_. Example images of this for a 2.1% w/w C_12_E_4_ film are shown in Figure S.7. At lower concentrations, the drop to below 90° did still occur, although usually over a longer time scale. We would like to draw attention to the fact that the first data point for the 6.4% w/w film is less than 90°. This is because the droplet, despite showing a high initial contact angle, was still distorted by inertia and was thus excluded. The next frame showed a contact angle of less than 90°.

While a decrease in θ below 90° is possible through the droplet receding due to evaporation, Figure S.7 shows contact angles on C_12_E_4_/PDMS films decreasing below 90° while the contact radii of the droplets increase. This demonstrates that the decrease in θ below 90° is not due to the droplet receding. Thus, we can deduce from the Young equation (eq 3) that if, initially, θ > 90° and γ_SG_ does not change, γ_SL_ must decrease to allow θ to drop below 90° while the droplet is advancing. As such, the change in contact angle must, at least, in part, be due to a change at the PDMS–water interface and not simply surfactant leaching into the water droplet, which would decrease γ_LG_.

This understanding of the Young equation demonstrates that the hydrophobic–hydrophilic switch in the surfactant/PDMS films following water contact is due to restructuring at the PDMS–water interface or the migration of surfactant from the bulk to the interface. Such a restructuring would be reasonably expected to produce a change in the roughness found using AFM when comparing films pre-immersion and post immersion. However, it is likely that the hydrophobic recovery occurs on too quick a time scale to be measured after a film has been removed from water.

When looking at the 4.2% w/w and 6.4% w/w films, the rate at which the contact angle decreases would be indicative of molecular reorientation being responsible. However, the 0.94% w/w, 2.1% w/w, and 3.2% w/w films showed a time lag before decreasing, demonstrating either a migration from a surfactant-rich buried interface or a longer time scale restructuring at the PDMS interface. This change from time-lag behavior to instantaneous hydrophilicity has been identified by Seo and Lee when doping PDMS with Triton X-100,^41^ and evidence of this may also be present in previous studies for the surfactants Tween 20, Brij 35,^40^ and trisiloxane ethoxylate^39^ in PDMS. However, there has been limited discussion of these regimes and the reasoning behind this change has attracted little attention. We can postulate that the time lag before the observed decrease in contact angle is dependent on the time taken for sufficient surfactant to migrate to the surface or, if already present, the time required for surfactant molecules to reorientate, changing from exposing their hydrophobic moieties to the water interface, to exposing hydrophilic chains. Figure 7 also shows a steady decline in contact angle for 0.24% w/w and 0.44% w/w C_12_E_4_, which is likely caused by the continuous migration of C_12_E_4_ from the bulk to the water/PDMS interface. Since there is no a priori reason why the rate of surface restructuring of surfactant should depend on bulk concentration, we postulate that the change in behavior is due to the rate of migration from the bulk to the surface.

The similarities of some of the temporal profiles are of great interest, with the 4.2% w/w and 6.4% w/w surfactant profiles being near-identical. This would suggest that, while the key processes in the hydrophobic–hydrophilic switch are concentration-dependent, there are distinct changes in behavior between regimes. We suggest the possibility that these regime changes are related to the phase behavior—increasing the concentration of C_12_E_4_ would result in a transition from one phase to two separate phases. This raises the question of whether the regime changes can be pinpointed to specific concentrations and whether this correlates with the phase behavior of the system.

**Figure 8:**
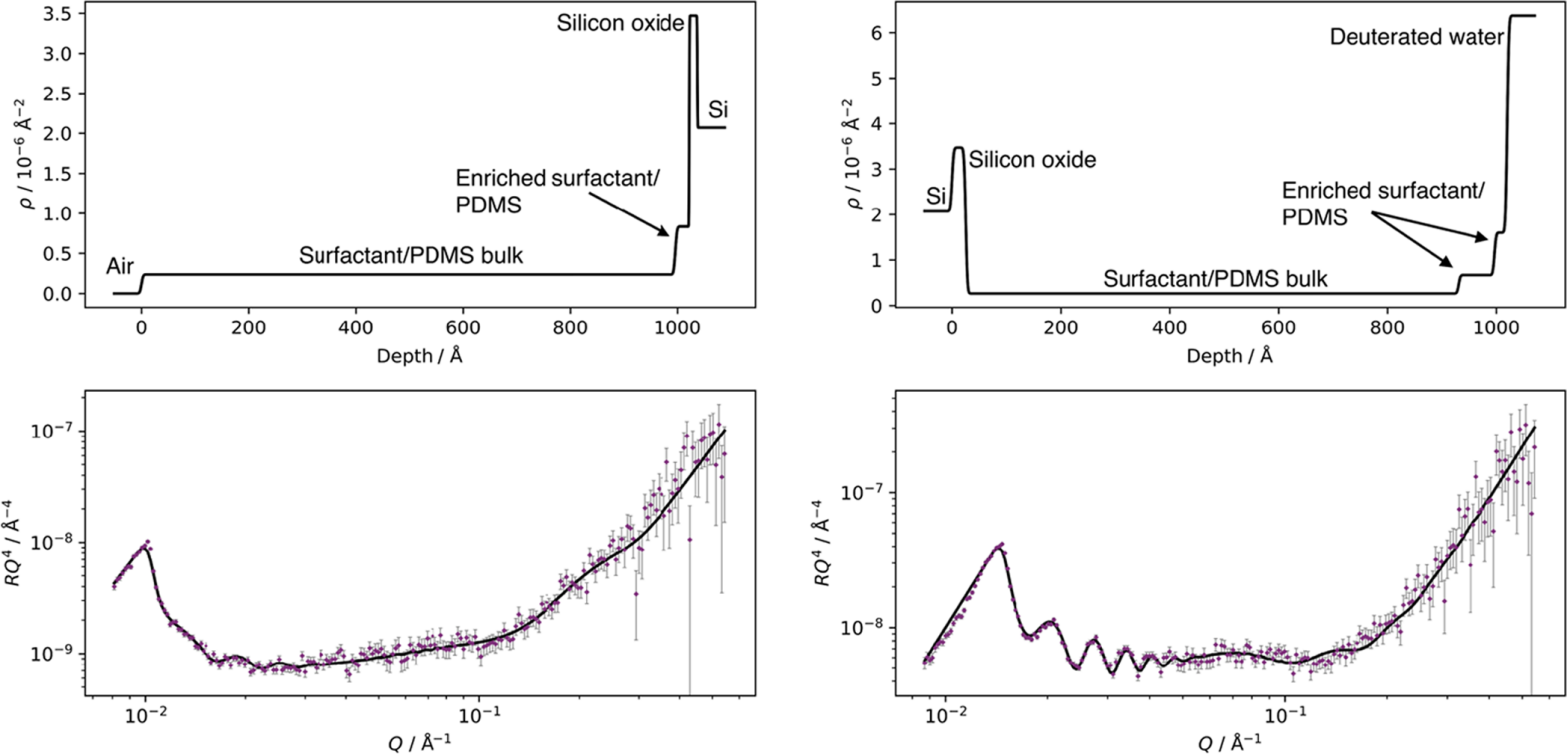
Neutron reflectivity of a cured thin film prepared using 4.1% w/w d_25_–C_12_E_4_ in PDMS against an air interface (left) and a D_2_O interface (right). Top: the optimized central SLD profiles used by MUSCtR for the fits. All three SLD profiles used by MUSCtR are shown for each sample in Figure S.8. Bottom: the reflectivity data and the fits corresponding to the optimized SLD profiles. The data are presented as *RQ*^4^ against *Q* to remove the *Q*^–4^ decay.

**Figure 9:**
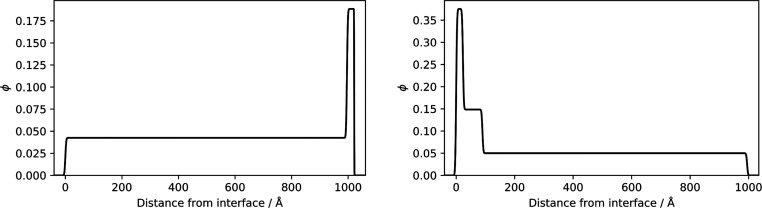
Vertical concentration profiles of d_25_–C_12_E_4_/PDMS films obtained from NR. ϕ is the volume fraction of d_25_–C_12_E_4_, and the distance is the depth from the air interface (left) and D_2_O interface (right).

To demonstrate the migration of C_12_E_4_ to the water interface following exposure, NR was performed on a thin 4.1% w/w d_25_–C_12_E_4_/PDMS film when exposed to an air interface and then exposed to a water interface. The reflectivity (*R*) data, fits, and SLD profiles are shown in Figure 8. Note that the silicon substrate (ρ_Si_ = 2.1 × 10^–6^ Å^–2^) and oxide layer (ρ_SiO_2__ = 3.5 × 10^–6^ Å^–2^) are at ∼1000 Å in the SLD profile in air but at ∼0 Å for the D_2_O interface due to the inverted geometry. The corresponding vertical concentration profiles of d_25_–C_12_E_4_ were then extracted using the equation
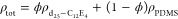
7where ϕ is the volume fraction of surfactant and ρ_tot_ is the measured SLD at a given depth. The vertical concentration profiles are shown in Figure 9.

Against an air interface, an appropriate fit could be obtained using only two layers of d_25_–C_12_E_4_ and PDMS: a bulk d_25_–C_12_E_4_/PDMS layer and an enriched layer of d_25_–C_12_E_4_ in PDMS. This enriched layer is at the silicon oxide interface. As such, NR supports the findings of NRA; the surfactant has a homogeneous vertical concentration profile near the air–PDMS interface. The small enrichment of surfactant at the silicon oxide interface is likely due to the surfactant reducing the interfacial energy between the nonpolar PDMS and the polar silicon oxide.

**Figure 10:**
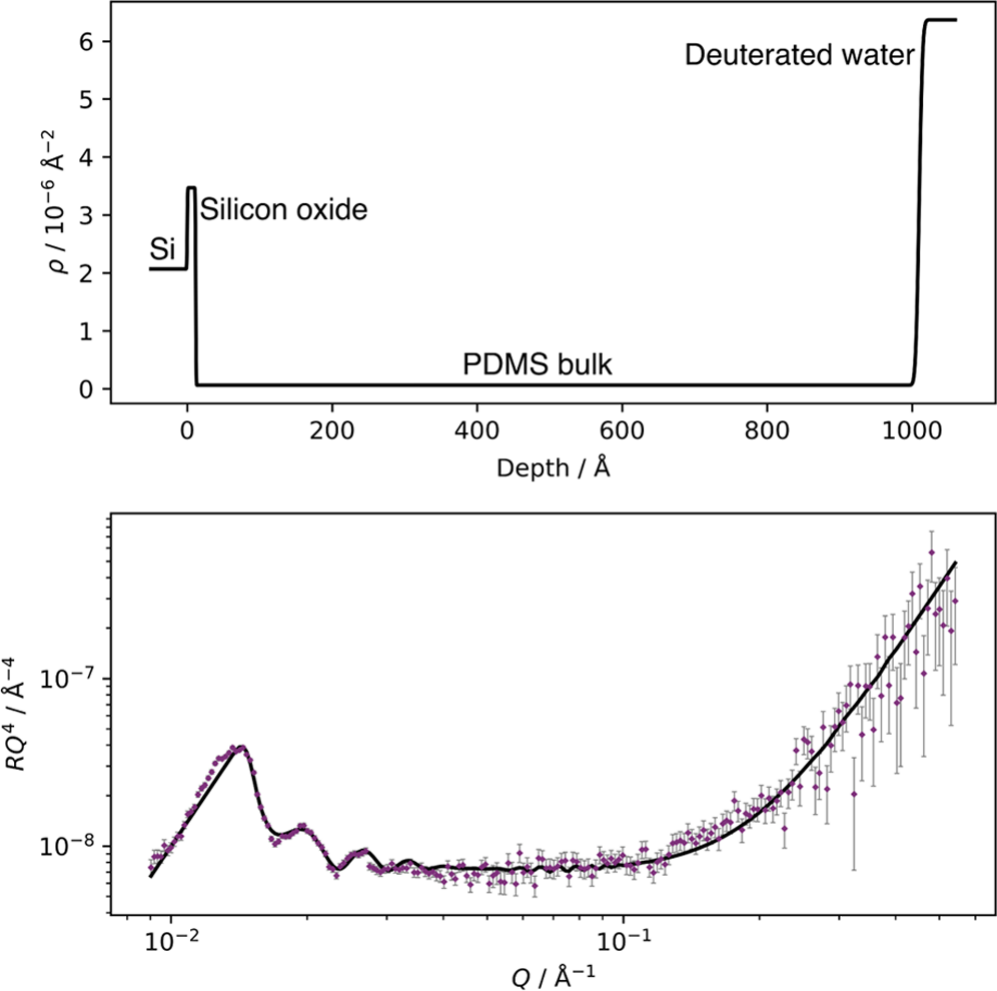
Neutron reflectivity of a cured thin film of PDMS against a D_2_O interface. Top: the optimized central SLD profile used by MUSCtR for the fit. All three SLD profiles used by MUSCtR are shown in Figure S.8. Bottom: the reflectivity data and the fit corresponding to the optimized SLD profile. The data are presented as *RQ*^4^ against *Q* to remove the *Q*^–4^ decay.

In contrast with the air interface, a good fit when exposed to D_2_O could not be obtained without enriched layers of d_25_–C_12_E_4_ near the water interface. While this increase in SLD could be due to the penetration of D_2_O into the PDMS matrix, such a layer was not needed to fit the reflectivity curve from a pure PDMS film against D_2_O (shown in Figure 10), suggesting that the layer is an enriched surfactant layer. This shows that, following exposure to water, a migration of surfactant from the PDMS bulk to the water–PDMS interface occurs. This supports the observations from WCA experiments.

### Impact of Curing on the Compatibility of C_12_E_4_/PDMS

**Figure 11:**
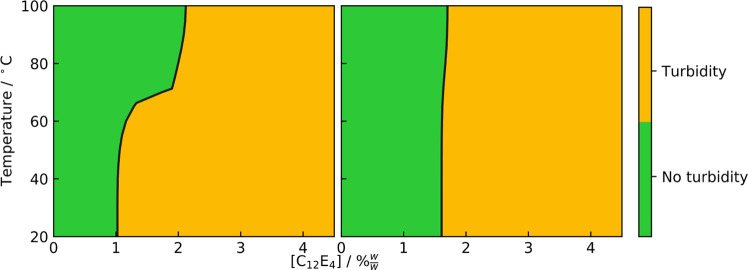
Compatibility contour maps from turbidity analysis of C_12_E_4_ in PDMS without cross-linker (left) and in cured PDMS using a 10:1 ratio of Sylgard 184 part A:part B (right). Example images showing the turbidity of samples are shown in Figure S.9.

Figure 11 shows the compatibility of C_12_E_4_ in PDMS up to ∼4.5% w/w, using a ratio of 10:1 Sylgard 184 part A:part B, when varying concentration and temperature. Turbidity arises from the phase separation of components into small domains, capable of scattering light. As such, a turbid sample was interpreted to be incompatible at the specified concentration of C_12_E_4_.

While the Flory–Rehner theory is often applied to consider compatibility of cross-linked systems, we are studying a system that has the polymer and solvent mixed before curing; thus, we are not observing swelling and the free energy of mixing will not be significantly perturbed by PDMS chain stretching. Work previously done by Clarke et al. has demonstrated that the phase behavior of branched polymer systems and polymer networks can be predicted using the Flory–Huggins Theory,^70^ and so the change in compatibility of C_12_E_4_ in PDMS during curing can instead be considered using Flory–Huggins. The free energy of mixing, Δ*G*_mix_, of a two-component mixture of a surfactant and PDMS can be expressed as^71^
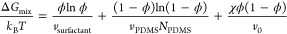
8where *k*_B_ is the Boltzmann constant, ϕ is the volume fraction of surfactant, χ is the interaction parameter of the two components, v_0_ is an arbitrary reference volume, *v*_surfactant_ and *v*_PDMS_ are the volume of monomer units of the surfactant and PDMS, respectively, and *N*_surfactant_ and *N*_PDMS_ are the respective degrees of polymerization of surfactant and PDMS, respectively. In a curing reaction, the degree of polymerization of PDMS increases. This results in a smaller magnitude of the second term on the right-hand side of the equation. The result is an increase in Δ*G*_mix_, meaning the compatibility is expected to decrease after curing, assuming the other terms remain unchanged. This is well established as the basis of reaction-induced phase separation,^72^ where polymerization can result in phase separation.

As expected, a higher concentration of surfactant results in an increase in turbidity for both cured and uncured samples. Interestingly, the turbidity in cured films was observed to begin between 1 and 2% w/w, which is similar to the concentration regime where we observed that the time-lag-type hydrophilization occurs in WCA. In addition, at ∼4% w/w, the samples became opaque. At this concentration in WCA, we observed a hydrophilicity regime change from the time-lag type to rapid hydrophilization.

However, what was not expected was that curing appeared to increase the compatibility of PDMS with C_12_E_4_ at low temperatures. Turbidity begins at ∼1% w/w C_12_E_4_ before curing and 2% w/w following curing at room temperature, a significant change. It is clear from eq 8 that the miscibility should decrease with increasing *N*_PDMS_, unless the effective interaction parameter decreases with increasing *N*_PDMS_.

To explain this, we can consider the free volume of the system. White et al. have shown that there is a strong correlation between the free volume of a polymer and the energy of the interaction between a molecule and its nearest neighbor, ε.^73^ As the free volume decreases, |ε| is seen to increase. Bell et al. have previously demonstrated acoustically that increasing the molecular weight of PDMS decreases the compressibility^74^ and thus the free volume, too. From this, we would expect curing PDMS to result in an increase in |ε_pp_|, the PDMS–PDMS interaction.

The interaction parameter can be defined as^75^
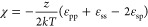
9where ε_ss_ and ε_sp_ are the surfactant–surfactant interaction and surfactant–PDMS interaction, respectively. An increase in |ε_pp_|, as we would expect to occur during curing, would consequently decrease χ if this brought it closer in value to |ε_ss_| due to |ε_sp_| scaling with |ε_pp_ – ε_ss_|. Looking at eq 8, if this decrease in χ sufficiently outweighs the effect of increasing *N*_PDMS_, Δ*G*_mix_ would decrease and thus increase the compatibility of the system during curing. To test this hypothesis, high precision data for the compressibility of all of the components would be required.

While the behavior of C_12_E_4_, including the critical micelle concentration, has been previously documented in water,^76,77^ here we have used PDMS as the solvent. Notably, the dodecyl chain antipathetic toward water is expected to be soluble in PDMS, with the reverse being true of C_12_E_4_’s oxyethylene chain. Thus, the self-assembly and phase behavior of C_12_E_4_ cannot be inferred from its behavior in water. To aid in elucidating the phase behavior and aggregation of C_12_E_4_ in PDMS, SANS can be used.

SANS was performed on d_25_–C_12_E_4_/Sylgard 184 part A mixtures at surfactant concentrations of 0.97% w/w, 2.8% w/w, 4.8% w/w, and 7.4% w/w. The part B cross-linker was then added at a ratio of 10:1 part A:part B and cured, followed by additional SANS measurements.

Two models have been used to fit the SANS data. The first model used was a two-power law^65^
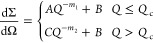
10where  is the scattering cross section, *Q* is the scattering wavevector, *Q*_c_ is the crossover point, *A* and *C* are the scaling coefficients for the low- and high-*Q* regions, respectively, *B* is the background intensity, and *m*_1_ and *m*_2_ are the power law exponents for the low- and high-*Q* regions, respectively. The second model used was a correlation length model^65^
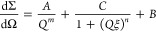
11where *A* and *C* are scaling factors, *B* is the background intensity, *m* is the Porod scattering exponent, *n* is the Lorentzian exponent, and ξ is the correlation length.

For systems without cross-linker, the two-power law (eq 10) was used for fitting since the model offered good fits in both the low-*Q* and the high-*Q* regions and due to the lack of any significant features, such as fringes or peaks. The two-power law was not deemed appropriate for SANS data from the cured samples at [d_25_–C_12_E_4_] = 0.87–4.4% w/w since the data sets show a broad shoulder at ∼0.03 Å^–1^. This feature is likely present due to the high correlation present in the cured network due to the presence of a cross-linked network; thus, a correlation length model (eq 11) was used for the cured samples.^62^

While the correlation length model could also be used to obtain adequate fits for samples without cross-linker, the uncertainty on ξ was significant, with fits obtainable for a range of ξ = 20–550 Å^–1^. Due to the absence of a distinct shoulder in , the high uncertainty in the fitting parameters, and the lack of a physical justification for a correlation length model (the uncured PDMS and d_25_–C_12_E_4_ mixtures were principally composed of unentangled linear chains), the correlation length model was not used for samples without cross-linker. The correlation length model was also deemed inappropriate for the 6.9% w/w d_25_–C_12_E_4_ cured sample due to insufficient curing and so was fit using a two-power law as well.

**Figure 12:**
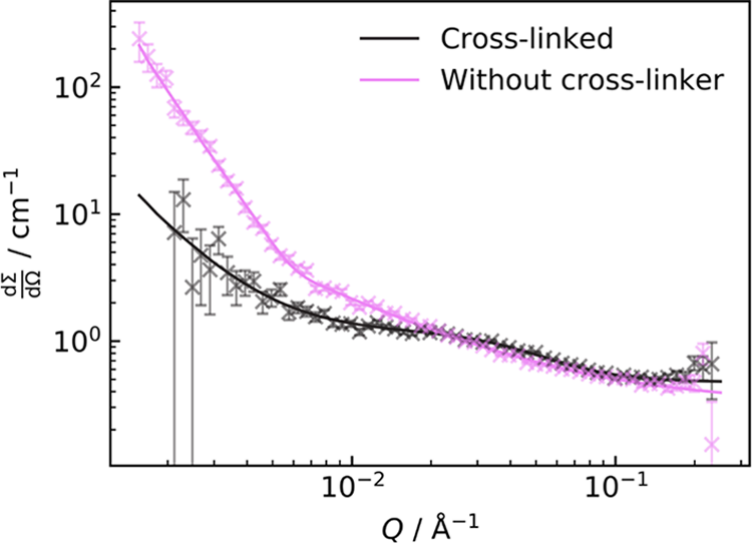
SANS curves of 0.97% w/w d_25_–C_12_E_4_ in PDMS without cross-linker and 0.87% w/w d_25_–C_12_E_4_ after curing. The change in concentration between the two data sets is a result of the addition of Sylgard 184 part B. The former was fit using a two-power law and the latter using a correlation length model.

**Figure 13:**
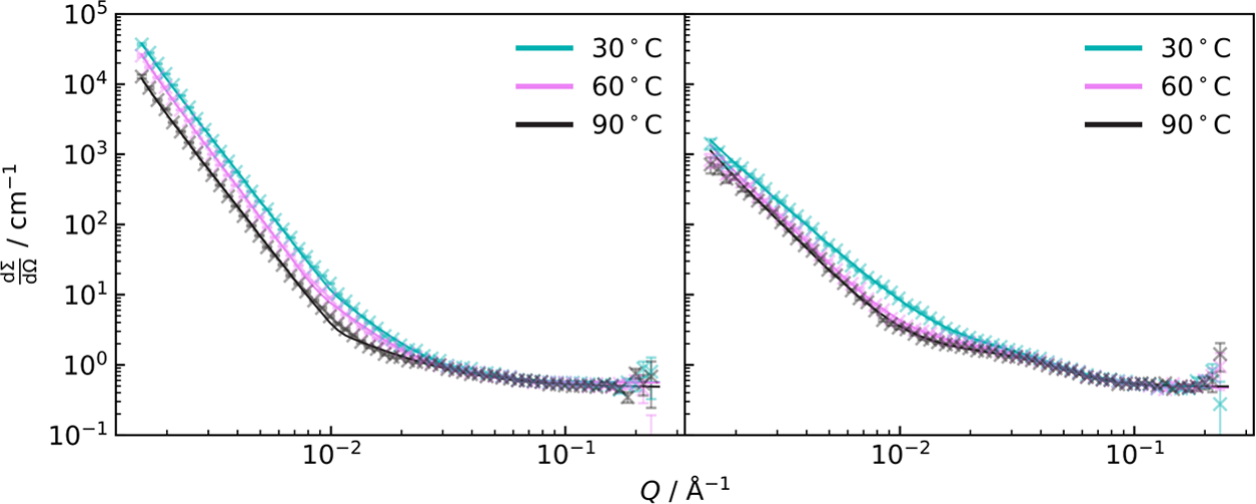
SANS curves of 4.8% w/w d_25_–C_12_E_4_ in PDMS without cross-linker (left) and 4.4% w/w d_25_–C_12_E_4_ following curing (right). The change in concentration between the two data sets is a result of the addition of Sylgard 184 part B. Curves on the left were fit using a two-power law, while for those on the right, a correlation length model was used.

SANS of ∼1% w/w and ∼5% w/w d_25_–C_12_E_4_ in PDMS are shown in Figures 12 and 13, respectively. Additional fits from this experiment are shown in Figure S.10.

The behavior of the 0.97% w/w mixture and the cured 0.87% w/w sample is distinctly different from that of the higher-concentration samples. Without cross-linker, the 0.97% w/w sample was fit using a low-*Q* Porod exponent of ∼3 and a high-*Q* exponent of 1. However, the higher surfactant concentrations all had respective exponents of ∼4 and 2. An exponent between 3 and 4 is indicative of a surface, with 4 being a smooth surface and 3 being a rough surface.^62^ As such, the higher concentrations exhibit phase separation into smooth domains, whereas the 0.97% w/w sample shows more weakly scattering rougher surfaces, indicating greater compatibility of the PDMS and surfactant.

For the high-*Q* exponents, *m*_2_ = 2 in the higher-concentration uncured mixtures could be attributed to dilute Gaussian chains, potentially surfactant molecules in the PDMS-dominant phase. However, the absence of a correlation between the integrated scattering intensity in the high-*Q* region and concentration of surfactant suggests that this is unlikely. Alternatively, this could be evidence of a surface-layer structure at the surfactant–PDMS interface.^62^ From this analysis of the samples without cross-linker, there does appear to be a change in phase behavior and structure between 0.97 and 2.8% w/w surfactants, which is consistent with turbidity results.

It is possible to estimate the specific surface area, *S*_T_, of phase-separated domains in the d_25_–C_12_E_4_/PDMS mixtures from the fits of the uncured samples when *m*_1_ = 4 using the low-*Q* region. This is done using Porod’s law,^78^ which can be expressed as
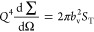
12where *b*_v_ is the contrast in scattering length densities of the two phases. This requires the assumption that the two phases are composed of pure d_25_–C_12_E_4_ and pure PDMS.

**Figure 14:**
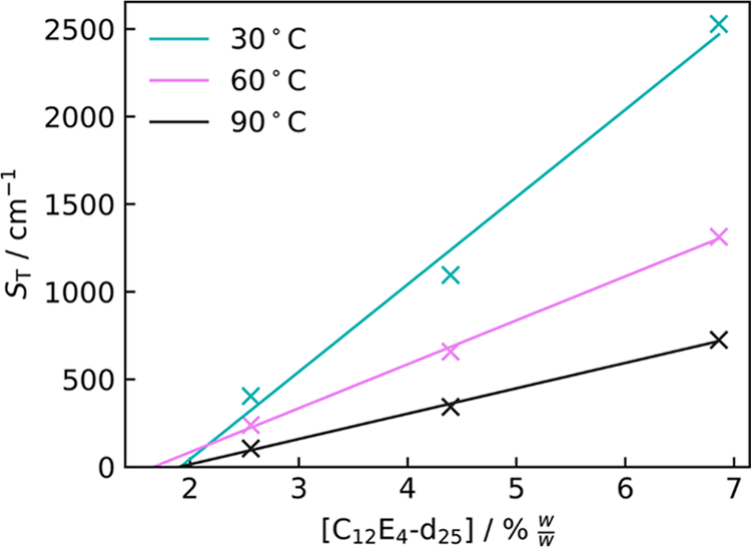
Estimated specific surfaces for samples of d_25_–C_12_E_4_/PDMS. *S*_T_ was found using Porod’s law.

**Figure 15:**
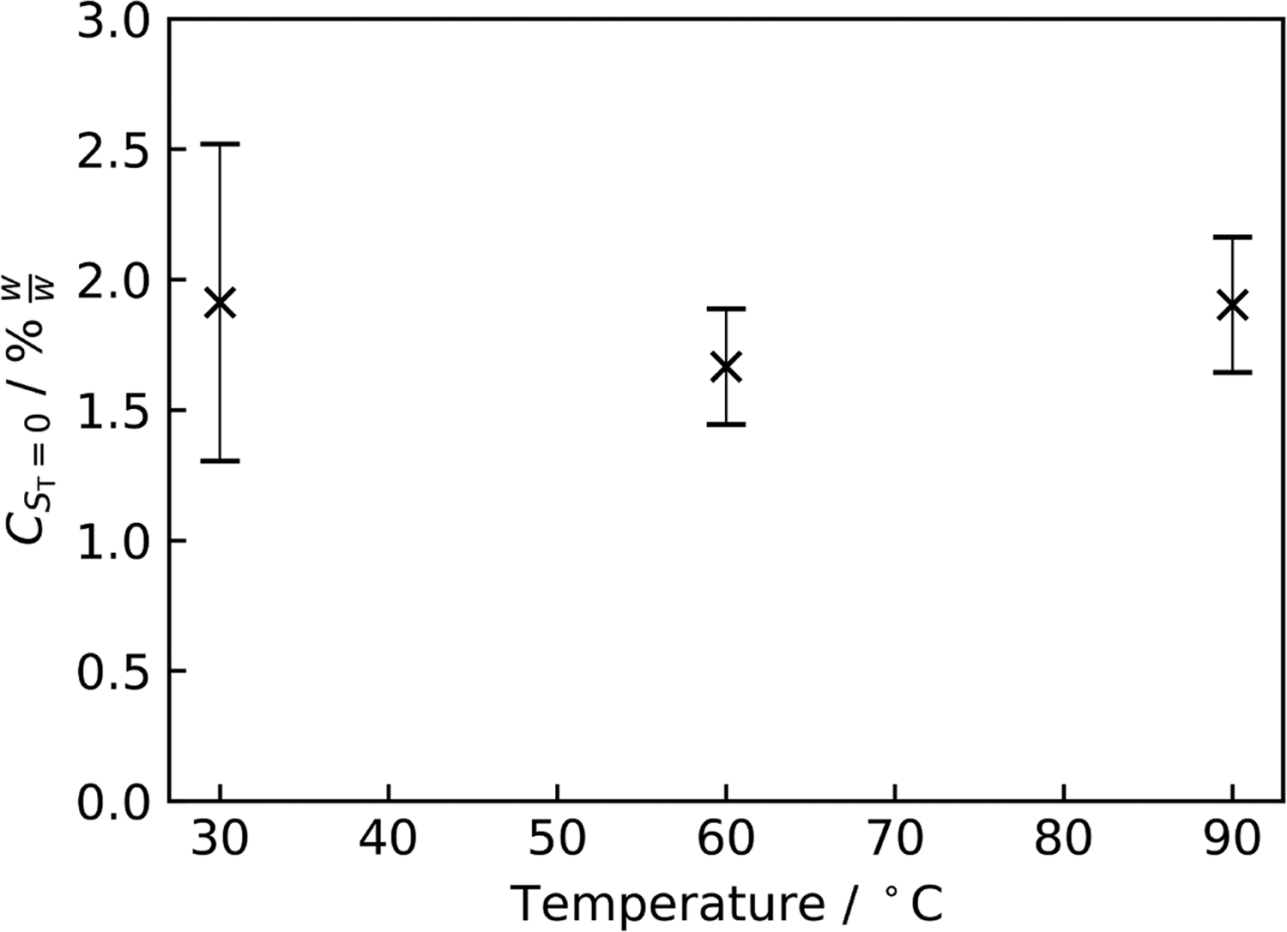
Extrapolated concentrations where *S*_T_ = 0 (*C*_*S*_T_ = 0_) for d_25_–C_12_E_4_ in PDMS.

By plotting *S*_T_ against concentration, as shown in Figure 14, we see the expected increase in *S*_T_ with the concentration of surfactant. *S*_T_ = 0 would be the point at which there is no longer an interface between the domains of the two phases, meaning there is only a single phase. By extrapolating linear fits for the concentration dependence of *S*_T_ to 0, we can estimate the concentration of d_25_–C_12_E_4_ at which phase separation occurs at each temperature. This is shown in Figure 15. At all three temperatures of 30, 60, and 90 °C, the limit of compatibility appears to be at ∼2% w/w. This further reaffirms the evidence for a phase behavior change being responsible for a hydrophilicity regime change and is in agreement with the observed turbidity data.

**Figure 16:**
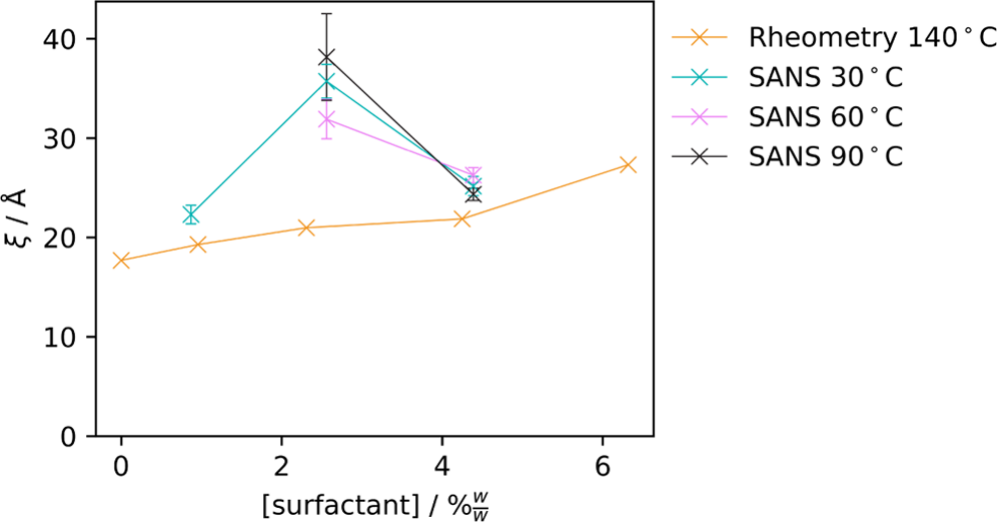
Estimated correlation length of surfactant/PDMS systems determined using SANS and rheometry. For rheometry experiments, the surfactant is C_12_E_4_, while for SANS experiments, it is d_25_–C_12_E_4_. The error bars on the rheometry measurements are too small to be seen on this scale.

For the cured samples, there is a shoulder in  at ∼0.03 Å^–1^. Similar features are being observed in other cross-linked systems^79−81^ due to the high correlation of such a matrix.^62^ The values for ξ obtained in the SANS fits for these samples likely behave as estimates for the average distance between cross-links or entanglements.^62^ To confirm this, we can compare these ξ values with estimates of the average distance between neighboring cross-links from the plateau moduli for 10:1 Sylgard 184 part A:part B samples in Figure 3 using eq 1. These results are shown in Figure 16.

While the values for ξ obtained from SANS and rheometry are not within error of one another, the similarity in magnitude of these values demonstrates that the broad “shoulder” in the SANS curves of the cured samples likely is the result of a correlated polymer matrix.

However, the low-*Q* Porod exponent of the fit for the 0.87% w/w d_25_–C_12_E_4_ cured sample was ∼2, whereas the higher concentrations showed *m* ≃ 3. This change in the small-angle scattering behavior between low and high concentrations shows that the low-*Q* features are likely not inherent to the PDMS matrix. The change in *m* is indicative of a change in surfactant/PDMS phase behavior between 0.87 and 2.6% w/w surfactants in cured PDMS. This supports the compatibility observations shown in Figure 11.

## Conclusions

We have demonstrated that the shear modulus of commercially available PDMS can be tuned by adjusting the concentration of the cross-linker used when curing and that the nonlinear rheology is different when using more or less than the concentration of cross-linker recommended by the manufacturer. The addition of the surfactant C_12_E_4_ does have a significant effect on the shear modulus of PDMS, which has not previously been reported to the best of the authors’ knowledge. We propose that this reduction is a result of the hygroscopicity of C_12_E_4_, resulting in water poisoning the catalyst in the curing reaction and may therefore be general to other amphiphilic or hydrophilic additives. Significantly, hydrophilic modification of PDMS may be difficult to achieve without some compromise in the mechanical properties, but we show that these effects can be readily quantified and to some extent mitigated by altering the quantities of the PDMS resin components.

The incorporation of C_12_E_4_, as seen with other surfactants, does result in the hydrophilization of the PDMS surface following exposure to water. We demonstrate that C_12_E_4_ is initially homogeneously distributed through the depth of a PDMS film, explaining the initial hydrophobicity of the film. Following water exposure, we have confirmed the presence of a hydrophobic–hydrophilic switch. Using NR, we have demonstrated that this switch is the result of a migration of initially homogeneously distributed surfactant from the PDMS bulk to the water interface, yielding an interfacial excess of C_12_E_4_. This surface enrichment is driven by a reduction in the PDMS–water interfacial energy. While the hydrophilicity can be increased by C_12_E_4_, we have observed limited potential for fine control over the surface energy, with three regimes of hydrophilicity identified, with one of the regime changes occurring due to a phase transition from one phase, to two distinct phases. Another factor to consider when adding surfactant is the increase in roughness—the efficacy of a fouling-release coating could be diminished by the increase in roughness, in spite of the decrease in hydrophobicity.

We have also presented the unexpected result that the C_12_E_4_/PDMS system becomes more compatible with curing. Since the Flory–Huggins theory shows that the entropic component of mixing becomes less favorable with curing, it would imply that the effective interaction parameter, χ, decreases. This could be due to an increase in the similarity of the average nearest-neighbor interaction energies of the PDMS and C_12_E_4_, ε_pp_, and ε_ss_ during curing.

## Supporting Information Available

In situ curing rheometry; MUSCtR description; Sylgard 184 possible reactions; NMR spectra; AFM images; NRA measurements; contact radius in WCA; NR SLD profiles; demonstration of turbidity; and SANS curves (PDF)
