# Supplementary material for: Tuning the Bulk and Surface Properties of PDMS Networks through Cross-Linker and Surfactant Concentration
Source: Macromolecules. 2021 Oct 6;54(20):9636–48. doi: 10.1021/acs.macromol.1c01600 (PMC8552438; doi:10.1021/acs.macromol.1c01600)
Supplement: Supplementary file 1 — ma1c01600_si_001.pdf [file ma1c01600_si_001.pdf]

Tuning the bulk and surface properties of PDMS networks through cross-linker and surfactant concentration

Matthew Litwinowicz,† Sarah Rogers,‡ Andrew Caruana,‡ Christy Kinane,‡ James Tellam,‡ and Richard Thompson*,†

†*Department of Chemistry, Durham University, Durham, DH1 3LE, United Kingdom*
‡*STFC ISIS Facility, Rutherford Appleton Laboratories, Chilton, Didcot, OX11 0QX, United Kingdom*

E-mail: r.l.thompson@durham.ac.uk

Supporting Information

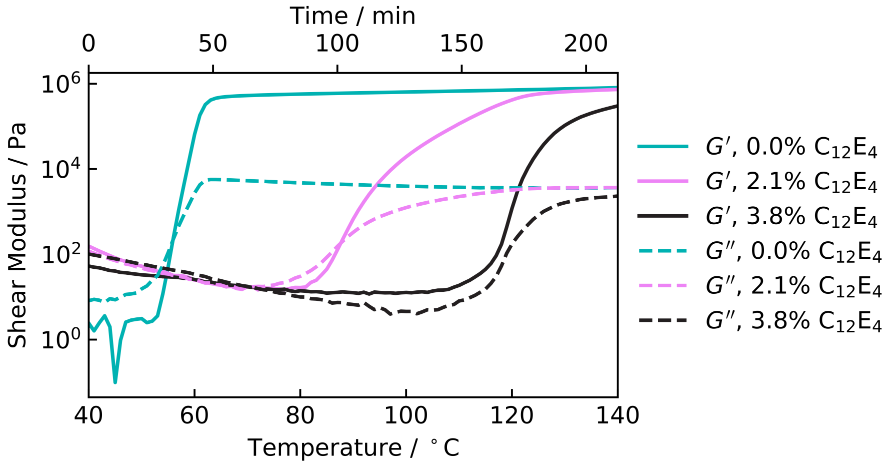

Figure S.1: The change in the shear moduli during a 0.47 °C min^{-1} temperature sweep of different concentrations of $C_{12}E_4$ in PDMS using a ratio of 5:1 Sylgard 184 part A : part B.

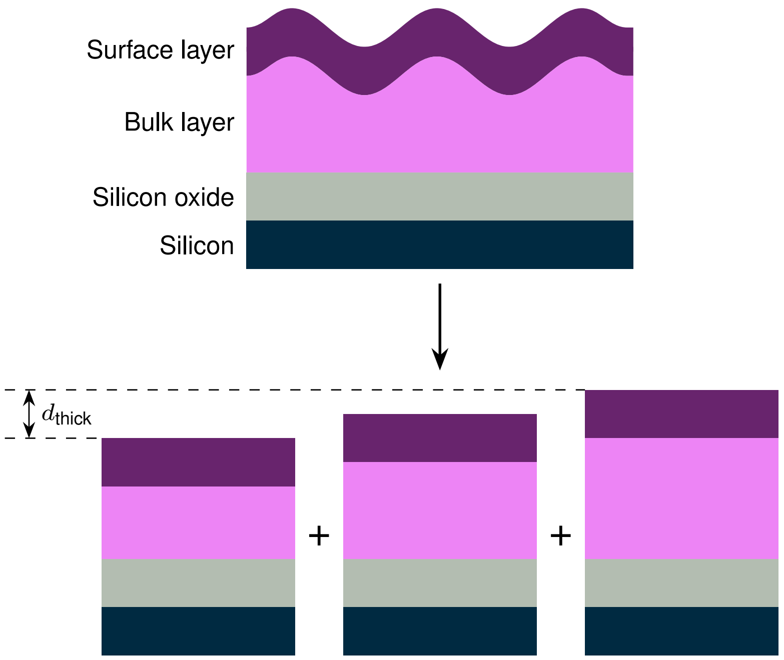

Figure S.2: The procedure used by MUSCtR to calculate reflectivity profiles for a film with a varying total thickness. A parameter, d_{thick}, characterises the variation in the thickness, t, of one layer of the model. The reflectivity is calculated for three cases: variable layer thickness $= t, t \pm {}^{d_{\text{thick}}}\!/_2$. A weighted average of the three curves is then taken, with the central profile given double the weighting of the thin and thick models. In this example, the bulk layer has been treated as having a variable thickness, thus MUSCtR uses the three profiles at the bottom to find the reflectivity curve.

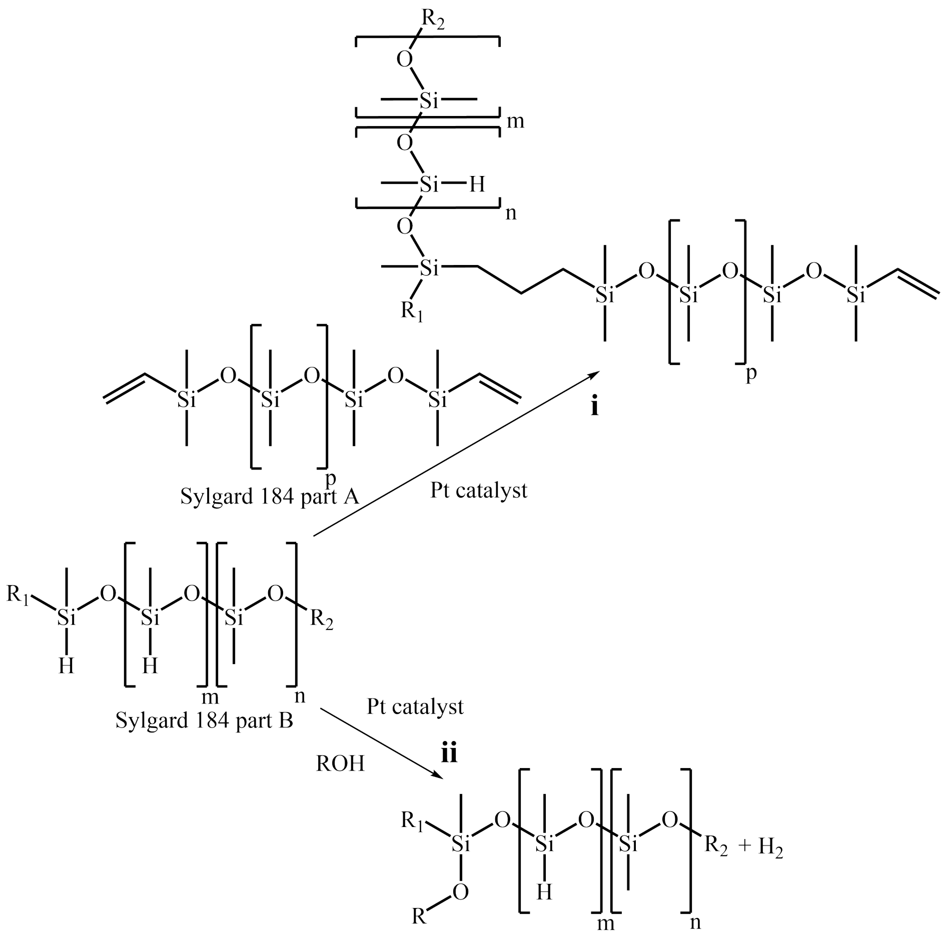

Figure S.3: Schemes of i) cross-linking reaction of Sylgard 184 (hydrosilylation) and ii) possible competing reaction of Sylgard 184 part B component with water and alcohols. R = H or alkyl, R_1 and R_2 = continued chain and terminus of poly(dimethylsiloxane-co-methylhydrosiloxane).

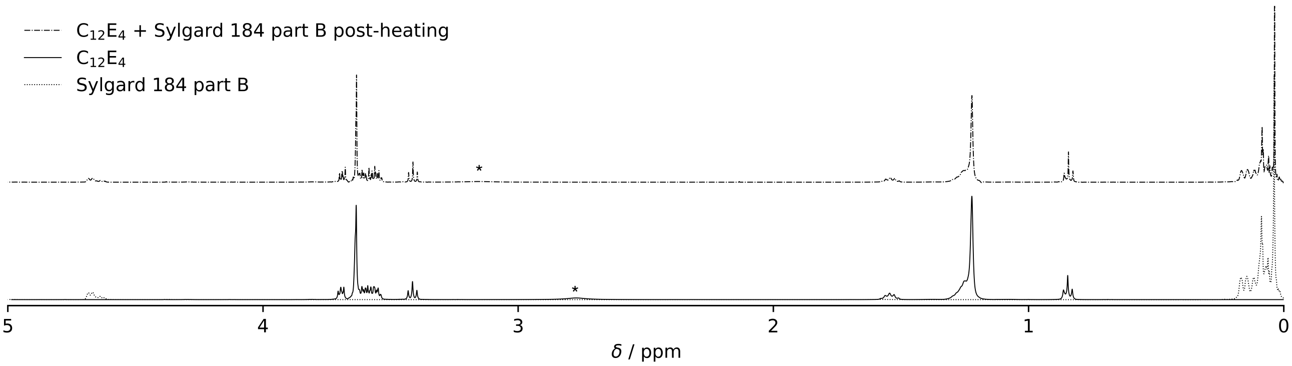

(a) ^{1}H Spectra

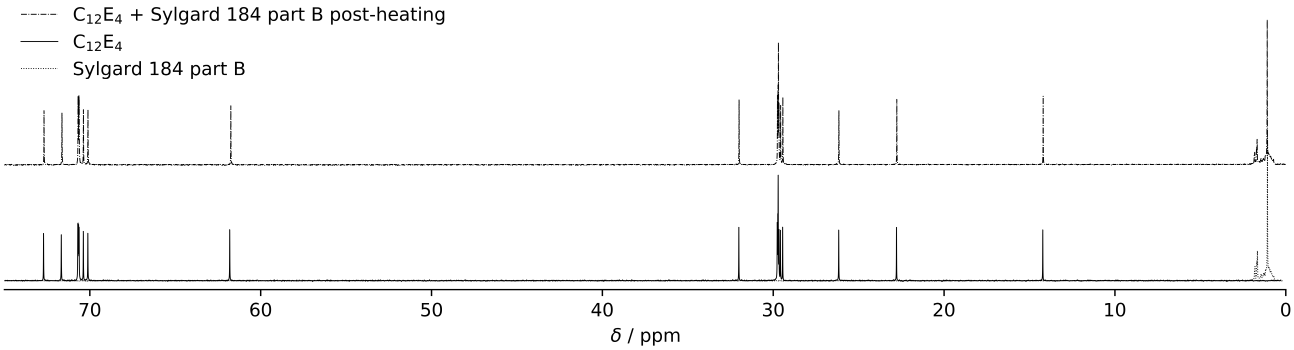

(b) ^{13}C Spectra

Figure S.4: ^{1}H and ^{13}C spectra of $C_{12}E_4$, Sylgard 184 part B, and a mixture of $C_{12}E_4$ and Sylgard 184 part B following heating at 100°C for one hour. There is no evidence of any new peaks to signify a new carbon or hydrogen environment in the mixture. "*" signifies the broad peaks corresponding to the −OH hydrogen environment in $C_{12}E_4$. PDMS and $C_{12}E_4$ were dissolved in $CDCl_3$ and spectra were recorded using a Bruker Avance III-HD-400 spectrometer.

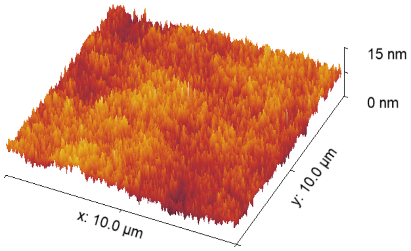

(a) 0.0%$^{w}/_{w}$ $C_{12}E_4$ before immersion

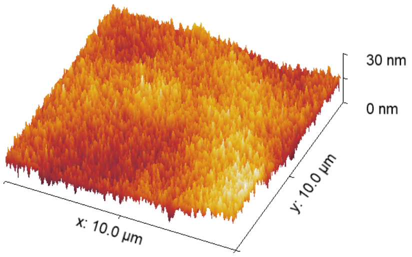

(b) 0.9%$^{w}/_{w}$ $C_{12}E_4$ before immersion

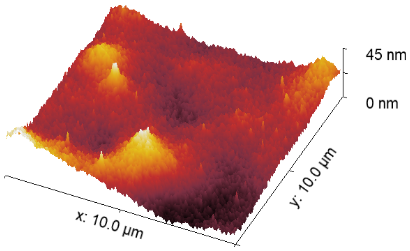

(c) 1.7%$^{w}/_{w}$ $C_{12}E_4$ before immersion

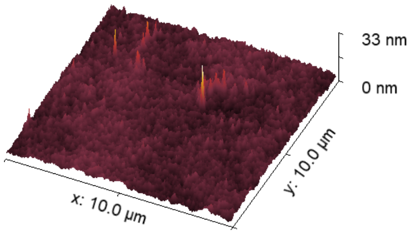

(d) 0.0%$^{w}/_{w}$ $C_{12}E_4$ after immersion

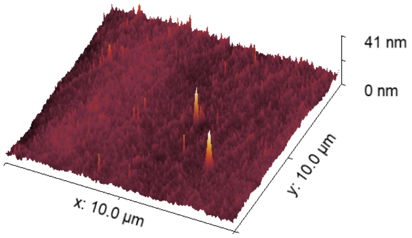

(e) 0.9%$^{w}/_{w}$ $C_{12}E_4$ after immersion

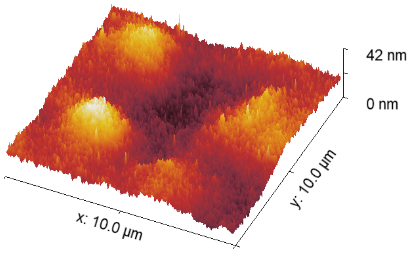

(f) 1.7%$^{w}/_{w}$ $C_{12}E_4$ after immersion

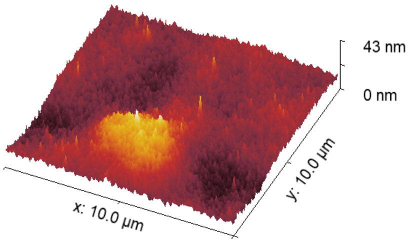

(g) 3.3%$^{w}/_{w}$ $C_{12}E_4$ before immersion

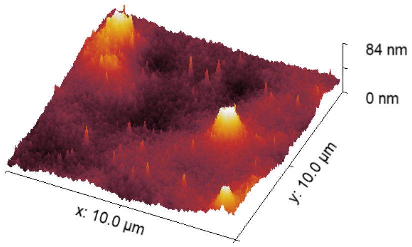

(h) 4.4%$^{w}/_{w}$ $C_{12}E_4$ before immersion

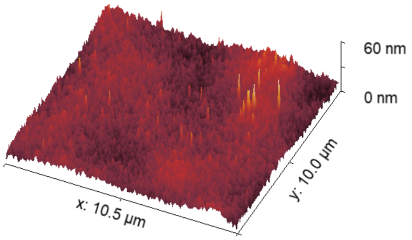

(i) 3.3%$^{w}/_{w}$ $C_{12}E_4$ after immersion

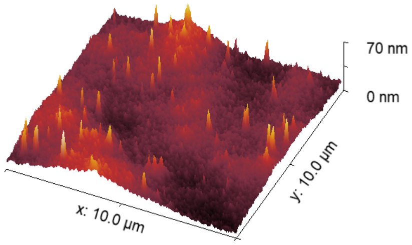

(j) 4.4%$^{w}/_{w}$ $C_{12}E_4$ after immersion

Figure S.5: AFM height maps of $C_{12}E_4$/PDMS films before and after 24 hours immersion in deionised water. Features are more prominent with more $C_{12}E_4$, but little change can be observed after immersion.

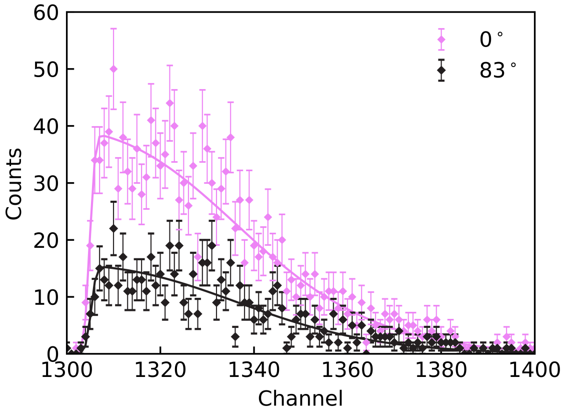

(a) 2.8%$^w/_w$ d_{25}−$C_{12}E_4$

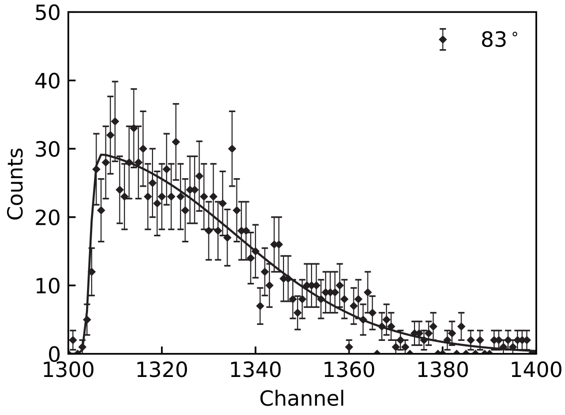

(b) 4.0%$^w/_w$ d_{25}−$C_{12}E_4$

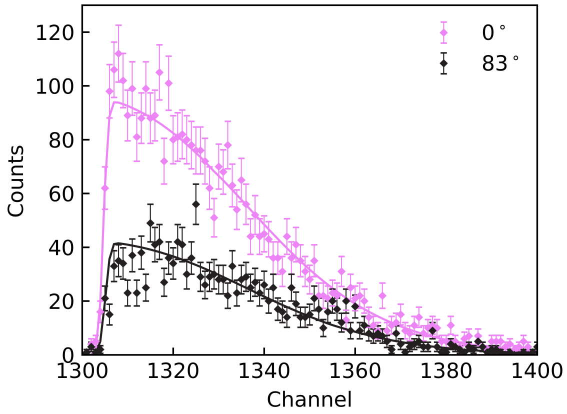

(c) 7.1%$^w/_w$ d_{25}−$C_{12}E_4$

Figure S.6: Data from NRA and the associated fits obtained using DataFurnace for d_{25}−$C_{12}E_4$/PDMS films with varying concentration of d_{25}−$C_{12}E_4$. The counts in the displayed channels are a result of the detection of a proton emitted by the nuclear reaction shown in eq 5. As the data is measured using counts, the error follows a Poisson distribution, yielding an error of $\sqrt{N}$ for a measurement of N counts.

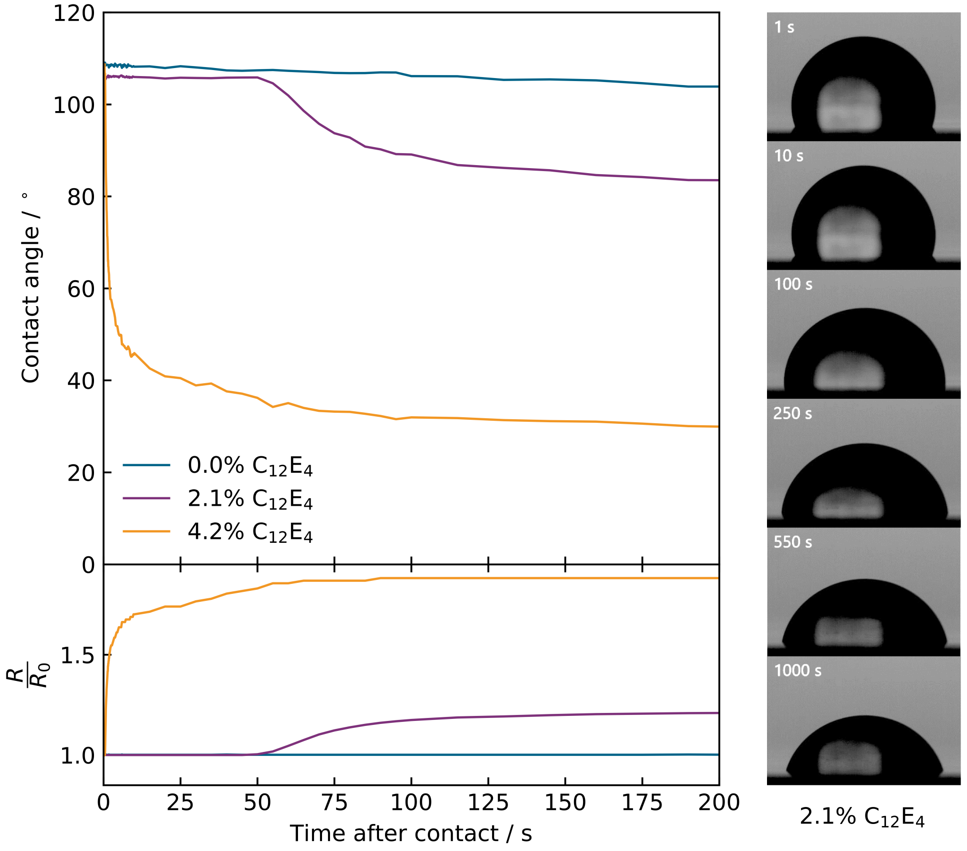

Figure S.7: Water contact angle analysis of $C_{12}E_4$/PDMS films. The water contact angles (top left) and normalised contact radii length (bottom left) are shown for 3 different concentration films during the first 200 s after making contact. The normalised contact radius was found by dividing the contact radius (R) by the initial contact radius (R_0). A selection of images is shown of the contact angle and contact radius of a water droplet changing on a 2.1%$^w/_w$ $C_{12}E_4$ film (right). All images are on the same scale.

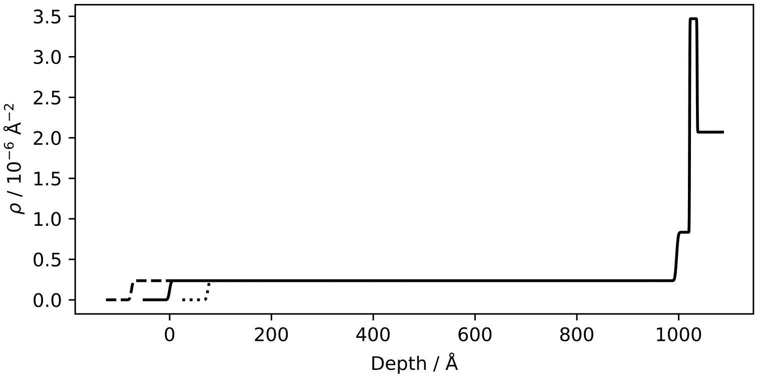

(a) 4.1%$^w/_w$ d_{25}−$C_{12}E_4$/PDMS film against air

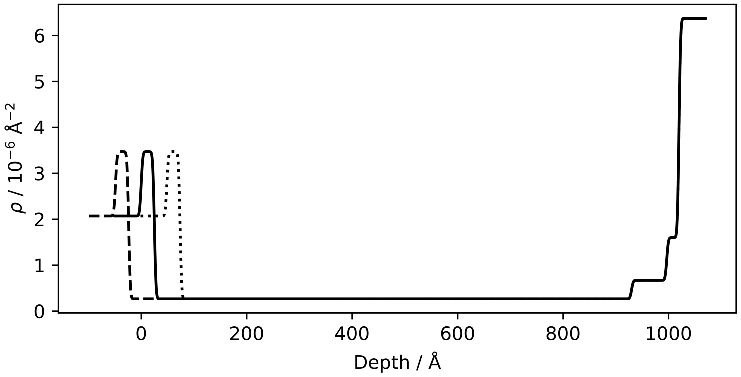

(b) 4.1%$^w/_w$ d_{25}−$C_{12}E_4$/PDMS film against D_2O

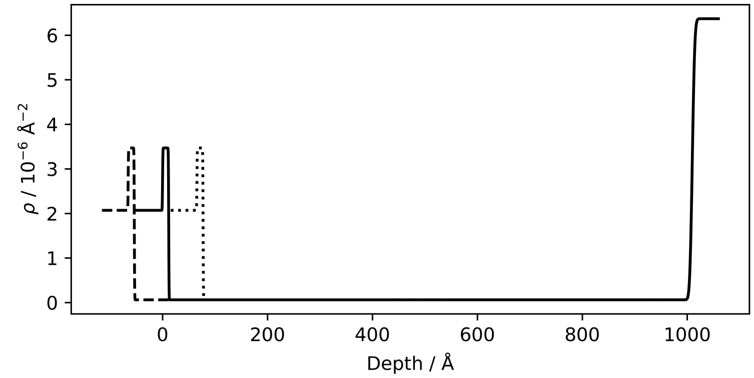

(c) PDMS film against D_2O

Figure S.8: The SLD profiles of three different films determined from fitting NR data using MUSCtR v1.4. For each plot, the thin, central, and thick models correspond to the dotted line, solid line, and dashed line, respectively. How these three profiles are used by MUSCtR is outlined in Figure S.2.

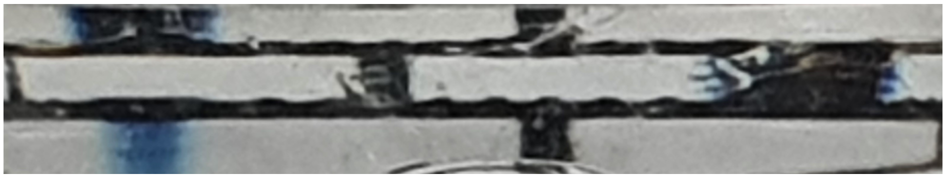

(a) $0\%\,{}^{w}/_{w}$ $C_{12}E_4$ in Sylgard 184: no turbidity

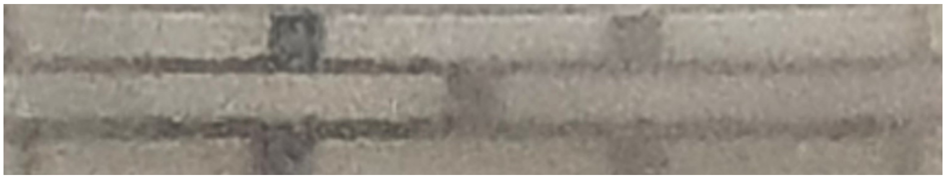

(b) $3.3\%\,{}^{w}/_{w}$ $C_{12}E_4$ in Sylgard 184: low turbidity

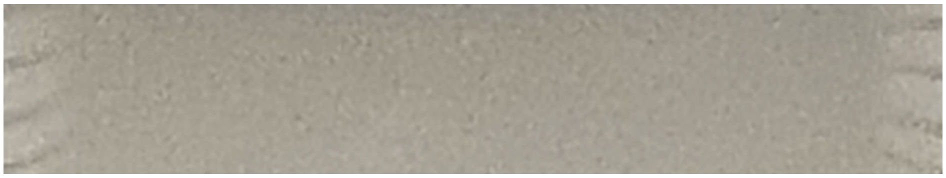

(c) $4.5\%\,{}^{w}/_{w}$ $C_{12}E_4$ in Sylgard 184: high turbidity

Figure S.9: The turbidity of $C_{12}E_4$/PDMS discs of 18 mm diameter. The discs were photographed in front of a black and white brick-pattern background. Turbidity can be observed to increase as the concentration of $C_{12}E_4$ increases.

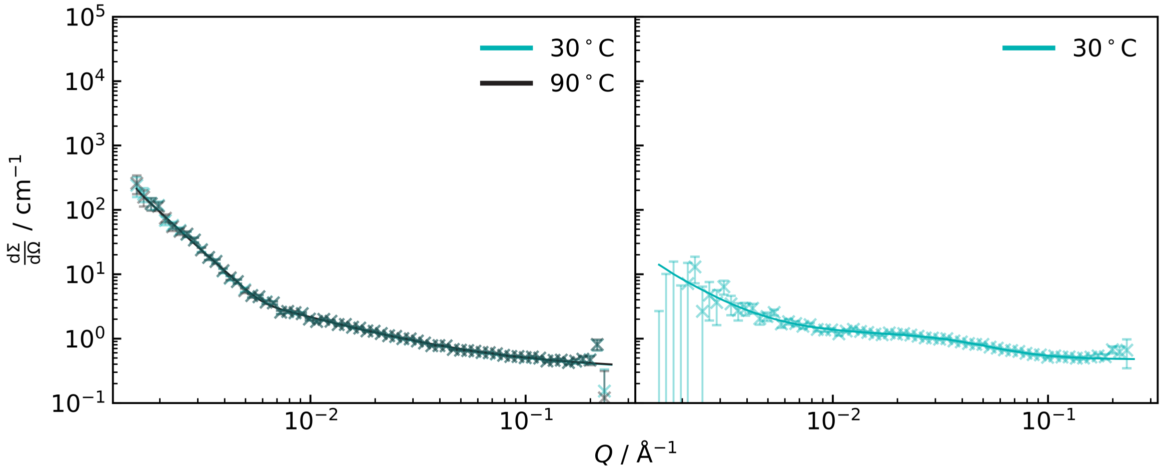

(a) 0.97%$^w/_w$ $d_{25}-C_{12}E_4$ in Sylgard 184 part A (left), 0.87%$^w/_w$ $d_{25}-C_{12}E_4$ in cured Sylgard 184 (right).

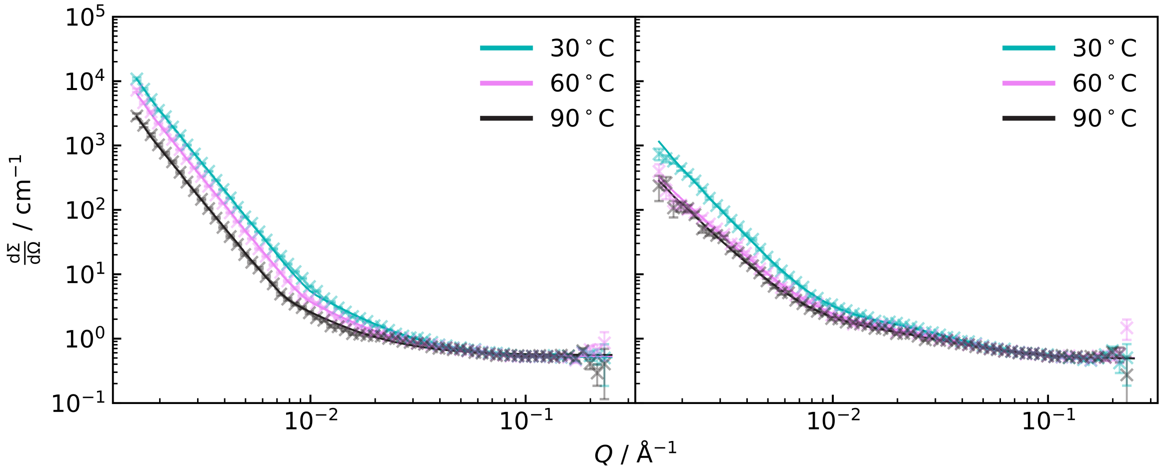

(b) 2.8%$^w/_w$ $d_{25}-C_{12}E_4$ in Sylgard 184 part A (left), 2.6%$^w/_w$ $d_{25}-C_{12}E_4$ in cured Sylgard 184 (right).

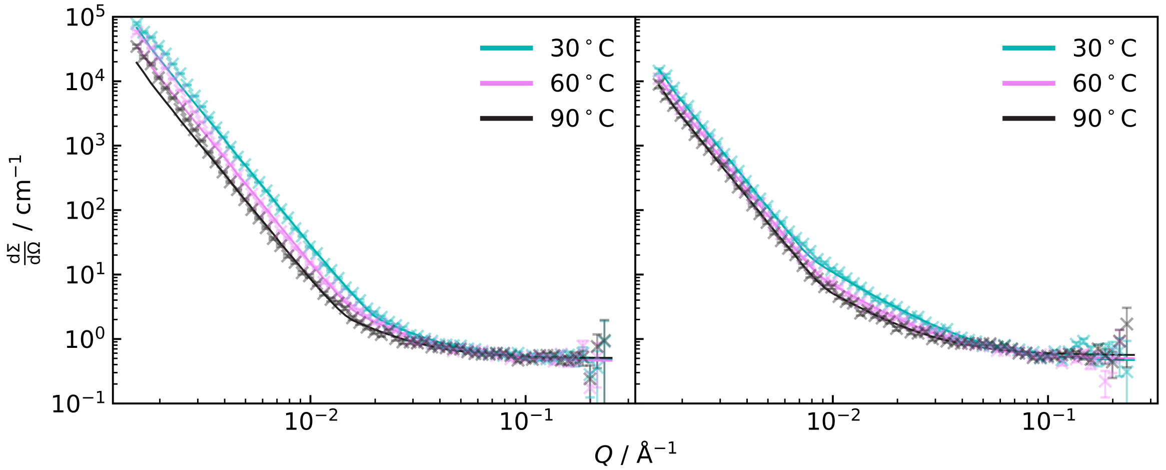

(c) 7.4%$^w/_w$ $d_{25}-C_{12}E_4$ in Sylgard 184 part A (left), 6.9%$^w/_w$ $d_{25}-C_{12}E_4$ in cured Sylgard 184 (right).

Figure S.10: SANS curves of different concentrations of $d_{25}-C_{12}E_4$ in PDMS without cross-linker (left) and following curing (right). The change in concentration between the two data sets is a result of the addition of Sylgard 184 part B.
